# Inter- and Intraobserver Variation in the Assessment of Preoperative Colostograms in Male Anorectal Malformations: An ARM-Net Consortium Survey

**DOI:** 10.3389/fped.2020.00571

**Published:** 2020-09-18

**Authors:** Paola Midrio, Iris A. L. M. van Rooij, Giulia Brisighelli, Aracelli Garcia, Maria Fanjul, Paul Broens, Barbara D. Iacobelli, Carlos Giné, Gabriele Lisi, Cornelius E. J. Sloots, Francesco Fascetti Leon, Anna Morandi, Herjan van der Steeg, Stefan Giuliani, Sabine Grasshoff-Derr, Martin Lacher, Ivo de Blaauw, Ekkehart Jenetzky

**Affiliations:** ^1^Pediatric Surgery Unit, Cà Foncello Hospital, Treviso, Italy; ^2^Department of Health Evidence, Radboud Institute for Health Sciences, Nijmegen, Netherlands; ^3^Department of Pediatric Surgery, Fondazione IRCCS Ca' Granda- Ospedale Maggiore Policlinico, Milan, Italy; ^4^Department of Pediatric Surgery, Chris Hani Baragwanath Hospital and University of Witwatersrand, Johannesburg, South Africa; ^5^Pediatric Surgery Unit, Doce de Octubre Universitary Hospital, Madrid, Spain; ^6^Pediatric Surgery Unit, Gregorio Marañón Universitary Hospital, Madrid, Spain; ^7^Division of Pediatric Surgery, Department of Surgery, University Medical Center Groningen, University of Groningen, Groningen, Netherlands; ^8^Department of Medical and Surgical Neonatology, Bambino Gesù Childrens Hospital, Rome, Italy; ^9^Department of Pediatric Surgery, Hospital Universitary Vall d'Hebron, Barcelona, Spain; ^10^Pediatric Surgery Unit, Department of Aging Science, University “G. d'Annunzio” of Chieti-Pescara, Chieti, Italy; ^11^Department of Pediatric Surgery, Erasmus Medical Center (MC)–Sophia Children's Hospital, Rotterdam, Netherlands; ^12^Pediatric Surgery Unit, University Hospital of Padua, Padua, Italy; ^13^Division of Pediatric Surgery, Department of Surgery, Radboudumc-Amalia Children's Hospital, Nijmegen, Netherlands; ^14^Department of Pediatric Surgery, St. George's University Hospitals NHS Foundation Trust, London, United Kingdom; ^15^Pediatric Surgery Unit, Buergerhospital and Clementine Kinderhospital, Frankfurt, Germany; ^16^Department of Pediatric Surgery, University of Leipzig, Leipzig, Germany; ^17^Department of Medicine, Faculty of Health, Witten/Herdecke University, Witten, Germany; ^18^Department of Child and Adolescent Psychiatry and Psychotherapy, University Medical Center of Johannes Gutenberg-University, Mainz, Germany

**Keywords:** anorectal malformation, urinary fistula, colostogram, surgery, ARM-net, multirater agreement, radiology, diagnostic study

## Abstract

**Aim:** Male patients with anorectal malformations (ARM) are classified according to presence and level of the recto-urinary fistula. This is traditionally established by a preoperative high-pressure distal colostogram that may be variably interpreted by different surgeons. The aim of this study was to evaluate the inter- and intraobserver variation in the assessment by pediatric surgeons of preoperative colostograms with respect to the level of the recto-urinary fistula.

**Materials and Methods:** Sixteen pediatric surgeons from 14 European centers belonging to the ARM-Net Consortium twice scored 130 images of distal colostograms taken in sagittal projection at a median age of 66 days of life (range: 4–1,106 days). Surgeons were asked to classify the fistula in bulbar, prostatic, bladder-neck, no fistula, and “unclear anatomy” example. Their assessments were compared with the intraoperative findings (kappa) for two scoring rounds with an interval of 6 months (intraobserver variation). Agreement among the surgeons' scores (interobserver variation) was also calculated using Krippendorff's alpha. A kappa over 0.75 is considered excellent, between 0.40 and 0.75 fair to good, and below 0.40 poor. Surgeons were asked to score the images in “poor” and “good” quality and to provide their years of experience in ARM treatment.

**Results:** Agreement between the image-based rating of surgeons and the intraoperative findings ranges from 0.06 to 0.45 (mean 0.31). Interobserver variation is higher (Krippendorff's alpha between 0.40 and 0.45). Years of experience in ARM treatment does not seem to influence the scoring. The mean intraobserver variation between the two rounds is 0.64. Overall, the quality of the images is considered poor. Images categorized as having a good quality result in a statistically significant higher kappa (mean: 0.36 and 0.37 in the first and second round, respectively) than in the group of bad-quality images (mean: 0.25 and 0.23, respectively).

**Conclusions:** There is poor agreement among experienced pediatric colorectal surgeons on preoperative colostograms. Techniques and analyses of images need to be improved in order to generate a homogeneous series of patients and make comparison of outcomes reliable.

## Introduction

The majority of patients born with anorectal malformations (ARM) have a fistula between the blind ending colon and the lower urinary tract in males and the genital apparatus in females. The classifications for ARM proposed over the years are based on specific anatomic characteristics, such as the level of the fistula in males and females (1–3). An appropriate classification is important as different levels of fistula correlate with different colorectal outcomes. The possibility to group patients based on similar characteristics is of great importance to uniform follow up and make different centers' series comparable (4). Indeed, one of the problems, when dealing with adolescents and adult patients, is to understand the original anatomic defect and preceding treatments in order to correctly address the complications (5–8).

In male patients, the presence and level of the urinary fistula is radiologically identified before surgical reconstruction by means of high-pressure distal colostogram. Based on this diagnostic study, male patients are labeled as bulbar, prostatic, bladder-neck, or no fistula, and surgery is planned accordingly (4). This sort of “label” defines the male patient throughout his follow-up and possible complications.

In spite of the fact that distal colostograms have been performed for many years all over the world and, very recently, specific papers have been published (9–11), images may be variably described and interpreted by radiologists and pediatric surgeons. There are multiple reasons for the different interpretations of the images, and these include heterogeneous techniques, positioning of the patient, type of contrast media, experience of the radiologist, presence of the surgeon during imaging, quality of images, and complexity of cases. For all the above mentioned variables, radiologists and/or surgeons can differently interpret the same radiologic study, and as a consequence, patients and corresponding clinical assessment during follow-up are not always comparable among different centers.

In 2010, a group of European clinicians founded the ARM-Net Consortium with the purpose to collaborate in genetic, epidemiological, and clinical research, to set up an anonymized registry of new ARM patients from the participating centers, and to improve the care for these patients (12, 13). More than 1900 ARM patients from 30 European pediatric surgical centers have been registered so far, thus making it the second largest cohort of ARM patients after the single center series of Peña and Levitt (14).

The purpose of this study was to collect radiological images of distal colostograms of male patients with ARM and circulate them among pediatric surgeons of the ARM-Net Consortium (15) in order to verify the concordance of interpretations to highlight pitfalls of images and assessment and, ultimately, to suggest indications for the proper execution of such an important diagnostic tool.

## Materials and Methods

### Study Design

The study is a diagnostic study on anonymized images (https://is.gd/colostogram), which assess the validity of surgical preoperative classification based on colostogram images. To exclude information bias, images were randomly mixed, and raters had no access to origin of images. The second rating was performed after 8.4 months. The study was approved by the Committee for Clinical Research of Cà Foncello Hospital (Treviso, Italy) with number 847/CE Marca.

### Colostogram Images

Sixteen pediatric surgeons from 14 ARM-Net Consortium centers participated in this study. Thirteen surgeons were asked to send images of distal colostograms of male patients who underwent surgery for ARM with either recto-bulbar, recto-prostatic, bladder-neck, or no fistula. The fistula was defined as bulbar when it was positioned at the level or below the external sphincter and prostatic when it was between the external sphincter and the bladder neck.

**Figure 1:**
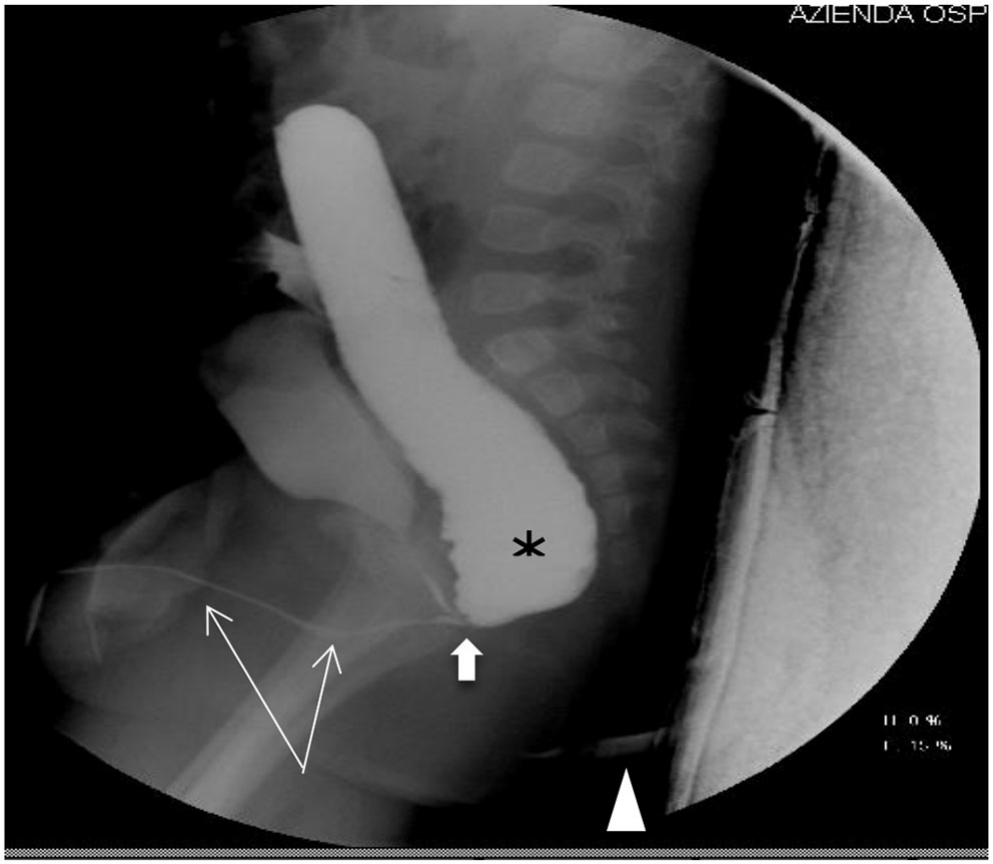
Recto-bulbar fistula: the distal colon is filled with contrast (asterisk), the entire urethra is visible (thin arrows), the recto-bulbar fistula is clearly recognizable (fat arrow), and the anal dimple is marked (arrowhead).

One image per patient, taken in sagittal projection and judged as the most representative of the patient, was submitted. Participants were instructed that, on an ideal study, the distal colon should be completely filled with contrast with the entire urethra and fistula clearly visible (Figure 1). All pictures were anonymized for patient and center before being sent to all participants. For each image, the final diagnosis of the type of fistula was provided based on the intraoperative finding as reported in the surgical report.

All surgeons were also asked to provide information on the age of the child at the time of imaging, the type of operation performed, and years of experience in ARM treatment and to justify why they decided that colostograms were considered of “poor” or “good” quality.

Images were judged as poor quality either because of insufficient contrast or too low of pressure or lacking a cysto-urethrogram. Surgeons found it difficult to classify images if any of the following situations occurred: the sacrum and/or distal urethra were not shown, the fistula was not clearly visible, there was a short distal colon or part of the urethra missing, the Foley catheter was in the urethra during imaging, rotated pictures, and missing perineal marker. Finally, some surgeons claimed they would have needed more pictures to reliably classify the image.

### Sample Size Calculation

According to Cantor (16), for all kappa-like agreement coefficients, the required number of subjects that needs to be included in a study depends on the relative acceptable error and the difference between the overall agreement probability and the chance-agreement probability. For the latter, we conservatively assumed 0. We defined a relative error of 20% and a probability difference of 0.5. We anticipated that the raters would agree about 50% of the time. Hence, we needed at least 100 images with a relative error of 20% (*n* = 44 with an error of 30%). With a lower inter-rater agreement of 0.4 (*n* = 156 or 69) or 0.3 at least 256 (20% relative error) or 123 images (30% relative error) would be the minimal size for valid results. Hence 130 images should give a valid answer.

### Data Management

Survey data were collected online using Research Electronic Data Capture (REDCap) tools hosted at the Department of Child and Adolescent Psychiatry and Psychotherapy, University Medical Center of the Johannes Gutenberg University Mainz, Germany (17). REDCap is a secure, web-based application designed to support data capture for research studies. Each surgeon received a password and independently scored all images, including their own, anonymously, and blinded to others. They were asked to classify the fistula as bulbar, prostatic, bladder-neck, no fistula, or unclear anatomy.

The surgeons scored the images twice with an interval of ~6 months between the two scoring rounds. In the second round of scoring, they also had to score the quality of the image into low (score = 1), medium (score = 2), or high quality (score = 3), or “cannot decide about quality” (score = 0). In both rounds, the information on the surgical report or the origin of the image was concealed, and in the second round, the voting from the first round was omitted to exclude information bias.

Each surgeon was also asked to provide information on their number of years of experience in scoring images of colostograms and the number of images scored per year.

### Statistical Analyses

To measure the level of agreement between the scoring of the surgeons and the intraoperative findings, the kappa was calculated for both the first and the second round of scoring. The differences between the scoring of the images in the first and second rounds for each surgeon is called the intraobserver variation (kappa). The mean intraobserver variation was calculated for the 16 pediatric surgeons. Finally, the agreement among the scoring of different surgeons was also calculated in both rounds as a measure of the interobserver variation. We used an online calculator for this measurement featuring the Krippendorff's alpha that also supported missing data in multiple raters (18, 19). All missing data remained in the data set although two images from the first round and three from the second round were excluded for this analysis because more than 85% of the ratings were missing.

We performed additional analyses by dividing the images based on quality to see whether high-quality images scored better than low-quality images. The average quality of each image was calculated. When the average was medium or higher (score ≥ 2), the quality of the image was categorized as good; if lower than medium (score < 2) it was categorized as bad. The mean kappa and intraobserver variation was compared between the group of images of good quality and those of bad quality using Student's *t*-test because of its normal distribution (Shapiro Wilk test). A kappa over 0.75 is usually considered to be excellent, between 0.40 and 0.75 is fair to good, and below 0.40 is poor. Statistical analyses were performed using SPSS Statistics, Version 22.0 for Windows (IBM SPSS Inc., Chicago, IL, USA).

## Results

In total, 135 distal colostograms were submitted by the participating centers. Five pictures were excluded due to evident low quality or as a second picture of the same patient. Therefore, 130 images were analyzed. The number of images per center ranged from 3 to 15.

The median age of patients at the time of the colostograms was 66 days of life (range: 4–1,106 days). The images were taken between the years 2000 and 2015 but mainly (70%) after 2010. Most surgeons (*n* = 11) had more than 10 years' experience in scoring images of ARM patients. Seven surgeons reported that they usually scored the images with a radiologist, five with other surgeons, three on their own, and one surgeon did not specify.

According to surgical reports, 53 patients had a recto-bulbar fistula (41%), 59 (45%) a recto-prostatic fistula, 15 (12%) a recto-bladder-neck fistula, and 3 (2%) an imperforate anus without fistula.

Agreement between the image-based rating of the surgeons and the surgical reports was low in both the first (kappa ranging from 0.06 to 0.43, mean 0.31) and second (0.14–0.45, mean 0.31) rounds (Table 1). The interobserver variation was generally higher but still quite low (Krippendorff's alpha of 0.40 and 0.45, respectively). The intraobserver variation between the first and second rounds with the same surgeon was higher with a mean kappa of 0.64 (SD: 0.11; range: 0.41–0.77). Years of experience in ARM treatment did not seem to influence the scoring.

**Table 1:** Kappa per surgeon for agreement between scoring and surgical reports in the first and second rounds and the intraobserver kappa between rounds 1 and 2.

**Surgeon no**.	**Years of experience in scoring**	**1st round**	**2nd round**	**Intraobserver variation 1st vs. 2nd round**
1	15	0.32	0.34	0.66
2	10	0.30	0.34	0.49
3	17	0.32	0.36	0.70
4	13	0.42	0.39	0.77
5	15	0.36	0.30	0.72
6	15	0.39	0.45	0.75
7	15	0.30	0.27	0.55
8	20	0.06	0.14	0.51
9	13	0.33	0.41	0.61
10	10	0.43	0.44	0.72
11	10	0.35	0.21	0.66
12	6	0.27	0.25	0.50
13	8	0.22	0.38	0.74
14	6	0.25	0.32	0.62
15	5	0.35	0.16	0.41
16	5	0.32	0.21	0.75
Mean (SD)	11.4 (4.6)	0.31 (0.09)	0.31 (0.10)	0.64 (0.11)

*SD, standard deviation*.

More than half (*n* = 69) of the total number of images were categorized as poor quality. These colostograms were mainly performed before 2010 (Table 2). The type of fistula did not substantially differ between images of good and bad quality. Agreement between surgeons' scoring and surgical reports was still low in the group of good-quality images (mean 0.36 and 0.37 in the first and second round, respectively), but significantly higher compared to the bad-quality images (mean 0.25 and 0.23, respectively). The intraobserver kappa was higher with the good-quality images, but the difference with the bad-quality images did not reach statistical significance. The same applied for the interobserver Krippendorff's alpha, being 0.46 for good- and 0.35 for bad-quality images in the first round and 0.49 and 0.41, respectively, in the second round.

**Table 2:** Difference between good- and bad-quality images in type of fistula, year of image, agreement on scoring the images, and intra- and interobserver variation.

	**Good-quality images** ***n* = 61**	**Bad-quality images** ***n* = 69**	***p*-value**
**Type of fistula intraoperatively decided**, ***n*** **(%)**			
Bulbar fistula	29 (48)	24 (35)	
Prostatic fistula	25 (41)	34 (49)	
Bladder-neck fistula	5 (8)	10 (15)	
No fistula	2 (3)	1 (1)	0.34^a^
**Year of image taken**			
<2010	14 (23)	25 (36)	
≥2010	47 (77)	44 (64)	0.10^a^
**Kappa, mean (SD)**			
With surgical report finding 1^st^ round	0.36 (0.10)	0.25 (0.10)	0.002^b^
With surgical report finding 2^nd^ round	0.37 (0.12)	0.23 (0.13)	0.002^b^
intraobserver (1^st^ vs. 2^nd^ round)	0.65 (0.13)	0.60 (0.16)	0.35^b^

*SD, standard deviation*,

a *Chi-square test*,

b *Student's t-test*.

## Discussion

This study shows poor agreement among experienced colorectal pediatric surgeons scoring the preoperative colostograms of male ARM patients even when the quality of images was assessed as medium or good. The reasons for poor agreement may be a combination of technical aspects, complexity of cases, and experience of both the radiologist and the pediatric surgeon in performing the study. To overcome the problem of personal interpretation of images, a more expensive and sophisticated technique, such as the pelvic-perineal MRI has been proposed (20). Conversely, in developing countries with limited access to contrast studies, the trans-perineal ultrasound-guided colostogram with saline has been proposed as an alternative method. However, that is an even more operator-dependent procedure (21, 22). Hosokawa et al. also proposes the use of ultrasound in combination with a voiding cystourethrogram in male neonates undergoing primary repair without colostomy with the aim to detect and locate the fistula as precisely as possible (23), and recently, the importance of the preoperative colostogram has been published (9–11). These last papers address both radiologists and pediatric surgeons and provide a series of technical details as well as the most common pitfalls that are extremely important and useful for correct clinical practice and fill the gap of knowledge highlighted in the present study.

From a technical point of view, when performing the colostogram, it is important to (a) fully distend the colon with the contrast medium, using a balloon-tip catheter with the inflated balloon inserted into the distal stoma in order to visualize the fistula and to avoid the false negative no fistula; (b) administer enough contrast to display both bladder and urethra and, therefore, determine the exact level of fistula; (c) mark the anal dimple in order to calculate the distance between the fistula and the perineum; (d) visualize the entire sacrum; and (e) correctly position the patient on sagittal view to avoid colon and bladder overprojection (11). The radiologists' experience in performing this contrast study may also play a key role and can only be acquired through the reporting of many cases. However, even in referral centers, the number of ARM male patients needing a colostogram is quite low; therefore, in each center, a team of pediatric radiologists and surgeons should be dedicated to these special cases, and diagnostic interpretations should be performed by multiple raters. Dedicated teams, in turn, should refer to standards of diagnosis provided by the international networks, such as the European Reference Network e-UROGEN (http://eurogen-ern.eu). It is the surgeons' responsibility to ensure the best for every ARM patient, and this includes rejecting suboptimal or, even worse, inadequate pictures for interpretation.

The importance of correctly classifying patients cannot be underestimated. When the anatomy of the patient is incorrectly defined before the surgical reconstruction, significant and severe consequences may occur. First, surgeons confident with laparoscopy may be induced to use this technique whenever the fistula is incorrectly described at a higher level, thus adding unnecessary known risks (24, 25). Second, based on incorrect preoperative information, the surgeon might look for the fistula at the wrong level along the urethra causing, again, avoidable complications. Finally, the outcome is expected to be different according to the different groups of patients, namely better, in terms of continence, for patients with bulbar fistulas and worse for those classified as prostatic and bladder-neck fistulas (26–29). Inadequate interpretation of the severity of malformations may lead to incorrect information being conveyed to parents and incorrect follow-up care. Moreover, the exchange of information about the outcome of patients with ARM among different centers might be incorrect and misleading if the wrong classifications are used.

A major strength of the study is the adequate sample size, available data set, and replicable evaluation for training purposes (https://is.gd/colostogram).

However, some limitations were also encountered. We considered the surgical report to be the gold standard when comparing the scoring of the colostogram. However, even this cannot be considered as a perfect standard because sometimes, during surgery, it may be difficult to exactly determine the level of the fistula. Moreover, it was clear from the scoring by the surgeons that a few of them tend to score more for one specific type of fistula, such as the bulbar fistula, and much less for the others. This may have biased the intraoperative finding as well. When looking at the scoring of the images, the agreement was somewhat higher for those images the surgeons provided themselves than for the total group of images, but it still did not exceed the excellent kappa border of 0.75 (data not shown). The fact that the intraobserver rate was 0.64 and, therefore, not perfect suggests that the surgeons had some doubts about the type of fistula and provided different answers at different times. A few centers provided almost exclusively good-quality images, but surgeons from these centers did not score better than others. In the setting of this study, the surgeon had to score the images alone although, in clinical practice, most surgeons perform the diagnostic assessment of the image together with other surgeons, and this might also have caused a bias. The fact that single images per patient were analyzed certainly limited the ability to accurately assess the location of the fistula along the urethra. In clinical practice, indeed, multiple images and views are reviewed to better understand the location of the fistula. This study was designed and performed by pediatric surgeons without involvement of radiologists. This contributed to making the study more uniform, but it might also have reduced the accuracy of the image assessment as radiological expertise was not taken into consideration. Finally, an explanation for the poor interobserver agreement could be that the location of the fistula does not always fall neatly into the three categories that were used as some fistulas occur in the transition areas of bulbar to prostatic urethra or prostatic urethra to bladder neck, making the precise determination of urethral location difficult to classify.

This is the first multicentric study that investigates the validity of a very diffuse practice: that is, the preoperative, high-pressure distal colostogram for male patients affected by ARM. The poor agreement among pediatric colorectal surgeons and the questions raised by the participants call for an improvement of images and analyses in order to provide more valid and valuable preoperative information. Moreover, it is important to generate a homogeneous series of patients and make comparison of outcomes among studies reliable. Training is necessary for pediatric surgeons to interpret the images as well as for radiologists to provide and interpret the radiological studies.

## Data Availability Statement

All datasets presented in this study are included in the article/Supplementary Material.

## Author Contributions

PM, IvR, and EJ designed the study, wrote, and reviewed the manuscript. ML, GB, and HS retrieved data and reviewed the manuscript. AG, MF, PB, BI, CG, GL, CS, FL, AM, SG, SG-D, and IdB retrieved data and searched the literature. All authors contributed to the article and approved the submitted version.

## Conflict of Interest

The authors declare that the research was conducted in the absence of any commercial or financial relationships that could be construed as a potential conflict of interest.

## References

[1] Stephens FD Smith ED. Anorectal Malformations in Children: Update 1988. Alan R. Liss, New York, NY: March of Dimes Birth Defects Foundation. (1988).

[2] Peña A. Anorectal malformations. Semin Pediatr Surg. (1995) 4:35–47. 7728507

[3] Holschneider A Hutson J Peña A Beket E Chatterjee S Coran A. Preliminary report on the international conference for the development of standards for the treatment of anorectal malformations. J Pediatr Surg. (2005) 40:1521–6. 10.1016/j.jpedsurg.2005.08.002 16226976

[4] Han Y Xia Z Guo S Yu X Li Z. Laparoscopically assisted anorectal pull-through versus posterior sagittal anorectoplasty for high and intermediate anorectal malformations: a systematic review and meta-analysis. PLoS ONE. (2017) 12:e0170421. 10.1371/journal.pone.0170421 28099464 PMC5242536

[5] Midrio P Battaglia S Urso E Castagnetti M Gamba P. Rectal adenocarcinoma in patients with anorectal malformations: report of two cases and a review of the literature. SpringerPlus. (2016) 5:1623. 10.1186/s40064-016-3263-5 27722042 PMC5030204

[6] de Blaauw I Midrio P Breech L Bischoff A Dickie B Versteegh HP. A Treatment of adults with unrecognized or inadequately repaired anorectal malformations: 17 cases of rectovestibular and rectoperineal fistulas. J Pediatr Adolesc Gynecol. (2013) 26:156–60. 10.1016/j.jpag.2012.12.003 23507006

[7] Giuliani S Midrio P De Filippo RE Vidal E Castagnetti M Zanon GF. Anorectal malformation and associated end-stage renal disease: management from newborn to adult life. J Pediatr Surg. (2013) 48:635–41. 10.1016/j.jpedsurg.2012.10.073 23480924

[8] Giuliani S Grano C Aminoff D Schwarzer N van de Vorle M Cretolle C. ARM-net Consortium. Transition of care in patients with anorectal malformations: consensus by the ARM-net consortium. J Pediatr Surg. (2017) 52:1866–72. 10.1016/j.jpedsurg.2017.06.008 28688794

[9] Abdalla WMA De La Torre L. The high pressure distal colostogram in anorectal malformations: technique and pitfalls. J Pediatr Surg. (2017) 52:1207–9. 10.1016/j.jpedsurg.2017.03.050 28381335

[10] Halleran DR Ahmad H Bates DG Vilanova-Sanchez A Wood RJ Levitt MA. A call to ARMs: accurate identification of the anatomy of the rectourethral fistula in anorectal malformations. J Pediatr Surg. (2019) 54:1708–10. 10.1016/j.jpedsurg.2019.04.010 31076157

[11] Kraus SJ Levitt MA Peña A. Augmented-pressure distal colostogram: the most important diagnostic tool for planning definitive surgical repair of anorectal malformations in boys. Pediatr Radiol. (2018) 48:258–69. 10.1007/s00247-017-3962-2 28840291

[12] de Blaauw I Wijers CH Schmiedeke E Holland-Cunz S Gamba P Marcelis CL. First results of a European multi-center registry of patients with anorectal malformations. J Pediatr Surg. (2013) 48:2530–5. 10.1016/j.jpedsurg.2013.07.022 24314198

[13] Wijers CH de Blaauw I Marcelis CL Wijnen RM Brunner H Midrio P. Research perspectives in the etiology of congenital anorectal malformations using data of the International Consortium on Anorectal Malformations: evidence for risk factors across different populations. Pediatr Surg Int. (2010) 26:1093–9. 10.1007/s00383-010-2688-0 20730541 PMC2962787

[14] Bischoff A Levitt MA Peña A. Update on the management of anorectal malformations. Pediatr Surg Int. (2013) 29:899–904. 10.1007/s00383-013-3355-z 23913263

[15] Jenetzky E van Rooij IA Aminoff D Schwarzer N Reutter H Schmiedeke E. The challenges of the european anorectal malformations-net registry. Eur J Pediatr Surg. (2015) 25:481–7. 10.1055/s-0035-1569149 26642384

[16] Cantor AB Sample size calculations for Cohen's Kappa. Psychol Methods. (1996) 1:150–3. 10.1037/1082-989X.1.2.150

[17] Harris PA Taylor R Thielke R Payne J Gonzalez N Conde JG. Research electronic data capture (REDCap) – a metadata-driven methodology and workflow process for providing translational research informatics support. J Biomed Inform. (2009) 42:377–81. 10.1016/j.jbi.2008.08.010 18929686 PMC2700030

[18] Geertzen J Inter-Rater Agreement With Multiple Raters and Variables. (2012) Retrieved from: https://nlp-ml.io/jg/software/ira/ (accessed May 18, 2017).

[19] Omoumi P Michoux N Larbi A Lacoste L Lecouvet FE Perlepe V. Multirater agreement for grading the femoral and tibial cartilage surface lesions at CT arthrography and analysis of causes of disagreement. Eur J Radiol. (2017) 88:95–101. 10.1016/j.ejrad.2016.12.026 28189216

[20] Thomeer MG Devos A Lequin M De Graaf N Meeussen CJ Meradji M. High resolution MRI for preoperative work-up of neonates with an anorectal malformation: a direct comparison with distal pressure colostography/fistulography. Eur Radiol. (2015) 25:3472–9. 10.1007/s00330-015-3786-0 26002129 PMC4636514

[21] Ekwunife OH Umeh EO Ugwu JO Ebubedike UR Okoli CC Modekwe VI eta al. Comparison of trans-perineal ultrasound-guided pressure augmented saline colostomy distension study and conventional contrast radiographic colostography in children with anorectal malformation. Afr J Paediatr Surg. (2016) 13:26–31. 10.4103/0189-6725.181703 27251520 PMC4955457

[22] Abdulkadir AY Abdur-Rahman LO Adesiyun OM. Nonfluoroscopic pressure colostography in the evaluation of genitourinary fistula of anorectal malformations: experience in a resource-poor environment. Pediatr Radiol. (2009) 39:132–6. 10.1007/s00247-008-1051-2 19020873

[23] Hosokawa T Yamada Y Tanami Y Sato Y Ishimaru T Tanaka Y. Comparison of diagnostic accuracy for fistulae at ultrasound and voiding cystourethrogram in neonates with anorectal malformation. Pediatr Radiol. (2019) 49:609–16. 10.1007/s00247-018-04339-4 30666353

[24] Bischoff A Martinez-Leo B Peña A Laparoscopic approach in the management of anorectal malformations Pediatr Surg Int. (2015) 31:431–7. 10.1007/s00383-015-3687-y 25725614

[25] Alam Sline Lawal TA Peña A Sheldon C Levitt MA. Acquired posterior urethral diverticulum following surgery for anorectal malformations. J Pediatr Surg. (2011) 46:1231–5. 10.1016/j.jpedsurg.2011.03.061 21683228

[26] Wigander H Nisell M Frenckner B Wester T Brodin U Öjmyr-Joelsson M. Quality of life and functional outcome in Swedish children with low anorectal malformations: a follow-up study. Pediatr Surg Int. (2019) 35:583–90. 10.1007/s00383-018-04431-8 30729983 PMC6456466

[27] Kyrklund K Pakarinen MP Taskinen S Rintala RJ. Bowel function and lower urinary tract symptoms in males with low anorectal malformations: an update of controlled, long-term outcomes. Int J Colorectal Dis. (2015) 30:221–8. 10.1007/s00384-014-2074-9 25435141

[28] Samuk I Bischoff A Hall J Levitt M Peña A. Anorectal malformation with rectobladder neck fistula: a distinct and challenging malformation. J Pediatr Surg. (2016) 51:1592–6. 10.1016/j.jpedsurg.2016.06.001 27345453

[29] Kyrklund K Pakarinen MP Rintala RJ. Long-term bowel function, quality of life and sexual function in patients with anorectal malformations treated during the PSARP era. Semin Pediatr Surg. (2017) 26:336–42. 10.1053/j.sempedsurg.2017.09.010 29110831

