# Supplementary material for: Inter- and Intraobserver Variation in the Assessment of Preoperative Colostograms in Male Anorectal Malformations: An ARM-Net Consortium Survey
Source: Front Pediatr. 2020 Sep 18;8:571. doi: 10.3389/fped.2020.00571 (PMC7531276; doi:10.3389/fped.2020.00571)
Supplement: Supplementary file 1 [file Data_Sheet_1.zip › CodeBook_Image2.pdf]

image2

Project Home | Project Setup | Online Designer | Data Dictionary | Codebook

Data Dictionary Codebook

13/06/2020 2:15pm

Collapse all instruments

#	Variable / Field Name	Field Label<br>*Field Note*	Field Attributes (Field Type, Validation, Choices, Calculations, etc.)
Instrument: **Urethral Fistula Images with quality** (urethral_fistula_images_with_quality)			Enabled as survey — Collapse
1	record_id	Record ID	text, Identifier
2	name	Section Header: *Now the second round starts, to assess the intra-rater reliability and to collect your opinion about the quality of these images. Before you start this exercise, be sure you have enough time, because it will take more than an hour to score them all and if you close your browser session before finishing, you may lose your former entries. You will not lose your entries if you click on the back button in the questionnaire. During a session the entries will be stored. Please look at your clock to measure the total time in minutes you need to rate these images. The needed time will be the last question. You are invited to rate about 130 images and assess the quality with a suggested urethral fistula of colostograms from 13 surgeons. On each page 20 images are presented. If the size of the images is not large enough, then try to reload the webpage or use another browser (e.g. Firefox). I recognized that occasionally the images appear to small to be rated without download. If you want to give feedback or a comment regarding an image or your rating, you can use the comment field below the image. Your feedback may include decision reasons, image quality or alternative solutions. This option may help you not to become frustrated and we could discuss problems later more easily. You can enter complete sentences, even though it does not appear on the screen. Please rate alone without influence of other colleagues! Good luck!*<br>Please write your name or email in this field:	text, Identifier
3	years_experience	Years of experience with scoring images of ARM patients:<br>*years*	text (number)
4	number_patients	Yearly number of ARM patients you score images from<br>*Not the number of ARM patients - just the number radiological scored*	text (number, Min: 0, Max: 100)
5	with_whom	With whom do you score images usually:	dropdown (autocomplete)<br>1 alone<br>2 together with other surgeons<br>3 together with radiologist<br>4 other circumstances
6	image001	Section Header: *Please decide which kind of fistula you assume in the following pictures.*<br>1. Picture	descriptive<br>Field Annotation: Please decide which kind of picture this is
7	answer001	Which type is picture 1	radio, Required<br>0 no fistula<br>1 bulbar fistula<br>2 prostatic fistula<br>3 bladderneck fistula<br>9 could not decide<br>Custom alignment: LH
8	quality_001	How do you rate the quality of this image?	radio, Required<br>3 Good quality<br>2 Medium quality<br>1 Low quality<br>0 cannot decide<br>Custom alignment: LH
9	comment_001	Comment to picture 1:	text<br>Field Annotation: Please decide which type you can see in this picture
10	image002	2. Picture	descriptive<br>Field Annotation: Please decide which kind of picture this is

11	answer002	Which type is picture 2	radio, Required<br>0 no fistula<br>1 bulbar fistula<br>2 prostatic fistula<br>3 bladderneck fistula<br>9 could not decide<br><br>Custom alignment: LH
12	quality_002	How do you rate the quality of this image?	radio, Required<br>3 Good quality<br>2 Medium quality<br>1 Low quality<br>0 cannot decide<br><br>Custom alignment: LH
13	comment_002	Comment to picture 2:	text<br>Field Annotation: Please decide which type you can see in this picture
14	image003	3. Picture	descriptive<br>Field Annotation: Please decide which kind of picture this is
15	answer003	Which type is picture 3	radio, Required<br>0 no fistula<br>1 bulbar fistula<br>2 prostatic fistula<br>3 bladderneck fistula<br>9 could not decide<br><br>Custom alignment: LH
16	quality_003	How do you rate the quality of this image?	radio, Required<br>3 Good quality<br>2 Medium quality<br>1 Low quality<br>0 cannot decide<br><br>Custom alignment: LH
17	comment_003	Comment to picture 3:	text<br>Field Annotation: Please decide which type you can see in this picture
18	image004	4. Picture	descriptive<br>Field Annotation: Please decide which type you can see in this picture
19	answer004	Which type is picture 4	radio, Required<br>0 no fistula<br>1 bulbar fistula<br>2 prostatic fistula<br>3 bladderneck fistula<br>9 could not decide<br><br>Custom alignment: LH
20	quality_004	How do you rate the quality of this image?	radio, Required<br>3 Good quality<br>2 Medium quality<br>1 Low quality<br>0 cannot decide<br><br>Custom alignment: LH
21	comment_004	Comment to picture 4:	text<br>Field Annotation: Please decide which type you can see in this picture
22	image005	5. Picture	descriptive<br>Field Annotation: Please decide which type you can see in this picture

23	answer005	Which type is picture 5	radio, Required<br>0 no fistula<br>1 bulbar fistula<br>2 prostatic fistula<br>3 bladderneck fistula<br>9 could not decide<br><br>Custom alignment: LH
24	quality_005	How do you rate the quality of this image?	radio, Required<br>3 Good quality<br>2 Medium quality<br>1 Low quality<br>0 cannot decide<br><br>Custom alignment: LH
25	comment_005	comment to picture 5	text<br>Custom alignment: RH
26	image006	6. Picture	descriptive<br>Field Annotation: Please decide which type you can see in this picture
27	answer006	Which type is picture 6	radio, Required<br>0 no fistula<br>1 bulbar fistula<br>2 prostatic fistula<br>3 bladderneck fistula<br>9 could not decide<br><br>Custom alignment: LH
28	quality_006	How do you rate the quality of this image?	radio, Required<br>3 Good quality<br>2 Medium quality<br>1 Low quality<br>0 cannot decide<br><br>Custom alignment: LH
29	comment_006	comment to picture 6	text<br>Custom alignment: RH
30	image007	7. Picture	descriptive<br>Field Annotation: Please decide which type you can see in this picture
31	answer007	Which type is picture 7	radio, Required<br>0 no fistula<br>1 bulbar fistula<br>2 prostatic fistula<br>3 bladderneck fistula<br>9 could not decide<br><br>Custom alignment: LH
32	quality_007	How do you rate the quality of this image?	radio, Required<br>3 Good quality<br>2 Medium quality<br>1 Low quality<br>0 cannot decide<br><br>Custom alignment: LH
33	comment_007	comment to picture 7	text<br>Custom alignment: RH
34	image008	8. Picture	descriptive<br>Field Annotation: Please decide which type you can see in this picture

35	answer008	Which type is picture 8	radio, Required<br>0 no fistula<br>1 bulbar fistula<br>2 prostatic fistula<br>3 bladderneck fistula<br>9 could not decide<br><br>Custom alignment: LH
36	quality_008	How do you rate the quality of this image?	radio, Required<br>3 Good quality<br>2 Medium quality<br>1 Low quality<br>0 cannot decide<br><br>Custom alignment: LH
37	comment_008	comment to picture 8	text<br>Custom alignment: RH
38	image009	9. Picture	descriptive<br>Field Annotation: Please decide which type you can see in this picture
39	answer009	Which type is picture 9	radio, Required<br>0 no fistula<br>1 bulbar fistula<br>2 prostatic fistula<br>3 bladderneck fistula<br>9 could not decide<br><br>Custom alignment: LH
40	quality_009	How do you rate the quality of this image?	radio, Required<br>3 Good quality<br>2 Medium quality<br>1 Low quality<br>0 cannot decide<br><br>Custom alignment: LH
41	comment_009	comment to picture 9	text<br>Custom alignment: RH
42	image010	10. Picture	descriptive<br>Field Annotation: Please decide which type you can see in this picture
43	answer010	Which type is picture 10	radio, Required<br>0 no fistula<br>1 bulbar fistula<br>2 prostatic fistula<br>3 bladderneck fistula<br>9 could not decide<br><br>Custom alignment: LH
44	quality_010	How do you rate the quality of this image?	radio, Required<br>3 Good quality<br>2 Medium quality<br>1 Low quality<br>0 cannot decide<br><br>Custom alignment: LH
45	comment_010	comment to picture 10	text<br>Custom alignment: RH
46	image011	11. Picture	descriptive<br>Field Annotation: Please decide which type you can see in this picture

47	answer011	Which type is picture 11	radio, Required<br>0 no fistula<br>1 bulbar fistula<br>2 prostatic fistula<br>3 bladderneck fistula<br>9 could not decide<br><br>Custom alignment: LH
48	quality_011	How do you rate the quality of this image?	radio, Required<br>3 Good quality<br>2 Medium quality<br>1 Low quality<br>0 cannot decide<br><br>Custom alignment: LH
49	comment_011	comment to picture 11	text<br>Custom alignment: RH
50	image012	12. Picture	descriptive<br>Field Annotation: Please decide which type you can see in this picture
51	answer012	Which type is picture 12	radio, Required<br>0 no fistula<br>1 bulbar fistula<br>2 prostatic fistula<br>3 bladderneck fistula<br>9 could not decide<br><br>Custom alignment: LH
52	quality_012	How do you rate the quality of this image?	radio, Required<br>3 Good quality<br>2 Medium quality<br>1 Low quality<br>0 cannot decide<br><br>Custom alignment: LH
53	comment_012	comment to picture 12	text<br>Custom alignment: RH
54	image013	13. Picture	descriptive<br>Field Annotation: Please decide which type you can see in this picture
55	answer013	Which type is picture 13	radio, Required<br>0 no fistula<br>1 bulbar fistula<br>2 prostatic fistula<br>3 bladderneck fistula<br>9 could not decide<br><br>Custom alignment: LH
56	quality_013	How do you rate the quality of this image?	radio, Required<br>3 Good quality<br>2 Medium quality<br>1 Low quality<br>0 cannot decide<br><br>Custom alignment: LH
57	comment_013	comment to picture 13	text<br>Custom alignment: RH
58	image014	14. Picture	descriptive<br>Field Annotation: Please decide which type you can see in this picture

59	answer014	Which type is picture 14	radio, Required<br>0 no fistula<br>1 bulbar fistula<br>2 prostatic fistula<br>3 bladderneck fistula<br>9 could not decide<br><br>Custom alignment: LH
60	quality_014	How do you rate the quality of this image?	radio, Required<br>3 Good quality<br>2 Medium quality<br>1 Low quality<br>0 cannot decide<br><br>Custom alignment: LH
61	comment_014	comment to picture 14	text<br>Custom alignment: RH
62	image015	15. Picture	descriptive<br>Field Annotation: Please decide which type you can see in this picture
63	answer015	Which type is picture 15	radio, Required<br>0 no fistula<br>1 bulbar fistula<br>2 prostatic fistula<br>3 bladderneck fistula<br>9 could not decide<br><br>Custom alignment: LH
64	quality_015	How do you rate the quality of this image?	radio, Required<br>3 Good quality<br>2 Medium quality<br>1 Low quality<br>0 cannot decide<br><br>Custom alignment: LH
65	comment_015	comment to picture 15	text<br>Custom alignment: RH
66	image016	16. Picture	descriptive<br>Field Annotation: Please decide which type you can see in this picture
67	answer016	Which type is picture 16	radio, Required<br>0 no fistula<br>1 bulbar fistula<br>2 prostatic fistula<br>3 bladderneck fistula<br>9 could not decide<br><br>Custom alignment: LH
68	quality_016	How do you rate the quality of this image?	radio, Required<br>3 Good quality<br>2 Medium quality<br>1 Low quality<br>0 cannot decide<br><br>Custom alignment: LH
69	comment_016	comment to picture 16	text<br>Custom alignment: RH
70	image017	17. Picture	descriptive<br>Field Annotation: Please decide which type you can see in this picture

71	answer017	Which type is picture 17	radio, Required<br>0 no fistula<br>1 bulbar fistula<br>2 prostatic fistula<br>3 bladderneck fistula<br>9 could not decide<br><br>Custom alignment: LH
72	quality_017	How do you rate the quality of this image?	radio, Required<br>3 Good quality<br>2 Medium quality<br>1 Low quality<br>0 cannot decide<br><br>Custom alignment: LH
73	comment_017	comment to picture 17	text<br>Custom alignment: RH
74	image018	18. Picture	descriptive<br>Field Annotation: Please decide which type you can see in this picture
75	answer018	Which type is picture 18	radio, Required<br>0 no fistula<br>1 bulbar fistula<br>2 prostatic fistula<br>3 bladderneck fistula<br>9 could not decide<br><br>Custom alignment: LH
76	quality_018	How do you rate the quality of this image?	radio, Required<br>3 Good quality<br>2 Medium quality<br>1 Low quality<br>0 cannot decide<br><br>Custom alignment: LH
77	comment_018	comment to picture 18	text<br>Custom alignment: RH
78	image019	19. Picture	descriptive<br>Field Annotation: Please decide which type you can see in this picture
79	answer019	Which type is picture 19	radio, Required<br>0 no fistula<br>1 bulbar fistula<br>2 prostatic fistula<br>3 bladderneck fistula<br>9 could not decide<br><br>Custom alignment: LH
80	quality_019	How do you rate the quality of this image?	radio, Required<br>3 Good quality<br>2 Medium quality<br>1 Low quality<br>0 cannot decide<br><br>Custom alignment: LH
81	comment_019	comment to picture 19	text<br>Custom alignment: RH
82	image020	20. Picture	descriptive<br>Field Annotation: Please decide which type you can see in this picture

83	answer020	Which type is picture 20	radio, Required<br>0 no fistula<br>1 bulbar fistula<br>2 prostatic fistula<br>3 bladderneck fistula<br>9 could not decide<br><br>Custom alignment: LH
84	quality_020	How do you rate the quality of this image?	radio, Required<br>3 Good quality<br>2 Medium quality<br>1 Low quality<br>0 cannot decide<br><br>Custom alignment: LH
85	comment_020	comment to picture 20	text<br>Custom alignment: RH
86	image021	Section Header:<br>21. Picture	descriptive<br>Field Annotation: Please decide which type you can see in this picture
87	answer021	Which type is picture 21	radio, Required<br>0 no fistula<br>1 bulbar fistula<br>2 prostatic fistula<br>3 bladderneck fistula<br>9 could not decide<br><br>Custom alignment: LH
88	quality_021	How do you rate the quality of this image?	radio, Required<br>3 Good quality<br>2 Medium quality<br>1 Low quality<br>0 cannot decide<br><br>Custom alignment: LH
89	comment_021	comment to picture 21	text<br>Custom alignment: RH
90	image022	22. Picture	descriptive<br>Field Annotation: Please decide which type you can see in this picture
91	answer022	Which type is picture 22	radio, Required<br>0 no fistula<br>1 bulbar fistula<br>2 prostatic fistula<br>3 bladderneck fistula<br>9 could not decide<br><br>Custom alignment: LH
92	quality_022	How do you rate the quality of this image?	radio, Required<br>3 Good quality<br>2 Medium quality<br>1 Low quality<br>0 cannot decide<br><br>Custom alignment: LH
93	comment_022	comment to picture 22	text<br>Custom alignment: RH
94	image023	23. Picture	descriptive<br>Field Annotation: Please decide which type you can see in this picture

95	answer023	Which type is picture 23	radio, Required<br>0 no fistula<br>1 bulbar fistula<br>2 prostatic fistula<br>3 bladderneck fistula<br>9 could not decide<br><br>Custom alignment: LH
96	quality_023	How do you rate the quality of this image?	radio, Required<br>3 Good quality<br>2 Medium quality<br>1 Low quality<br>0 cannot decide<br><br>Custom alignment: LH
97	comment_023	comment to picture 23	text<br>Custom alignment: RH
98	image024	24. Picture	descriptive<br>Field Annotation: Please decide which type you can see in this picture
99	answer024	Which type is picture 24	radio, Required<br>0 no fistula<br>1 bulbar fistula<br>2 prostatic fistula<br>3 bladderneck fistula<br>9 could not decide<br><br>Custom alignment: LH
100	quality_024	How do you rate the quality of this image?	radio, Required<br>3 Good quality<br>2 Medium quality<br>1 Low quality<br>0 cannot decide<br><br>Custom alignment: LH
101	comment_024	comment to picture 24	text<br>Custom alignment: RH
102	image025	25. Picture	descriptive<br>Field Annotation: Please decide which type you can see in this picture
103	answer025	Which type is picture 25	radio, Required<br>0 no fistula<br>1 bulbar fistula<br>2 prostatic fistula<br>3 bladderneck fistula<br>9 could not decide<br><br>Custom alignment: LH
104	quality_025	How do you rate the quality of this image?	radio, Required<br>3 Good quality<br>2 Medium quality<br>1 Low quality<br>0 cannot decide<br><br>Custom alignment: LH
105	comment_025	comment to picture 25	text<br>Custom alignment: RH
106	image026	26. Picture	descriptive<br>Field Annotation: Please decide which type you can see in this picture

107	answer026	Which type is picture 26	radio, Required<br>0 no fistula<br>1 bulbar fistula<br>2 prostatic fistula<br>3 bladderneck fistula<br>9 could not decide<br><br>Custom alignment: LH
108	quality_026	How do you rate the quality of this image?	radio, Required<br>3 Good quality<br>2 Medium quality<br>1 Low quality<br>0 cannot decide<br><br>Custom alignment: LH
109	comment_026	comment to picture 26	text<br>Custom alignment: RH
110	image027	27. Picture	descriptive<br>Field Annotation: Please decide which type you can see in this picture
111	answer027	Which type is picture 27	radio, Required<br>0 no fistula<br>1 bulbar fistula<br>2 prostatic fistula<br>3 bladderneck fistula<br>9 could not decide<br><br>Custom alignment: LH
112	quality_027	How do you rate the quality of this image?	radio, Required<br>3 Good quality<br>2 Medium quality<br>1 Low quality<br>0 cannot decide<br><br>Custom alignment: LH
113	comment_027	comment to picture 27	text<br>Custom alignment: RH
114	image028	28. Picture	descriptive<br>Field Annotation: Please decide which type you can see in this picture
115	answer028	Which type is picture 28	radio, Required<br>0 no fistula<br>1 bulbar fistula<br>2 prostatic fistula<br>3 bladderneck fistula<br>9 could not decide<br><br>Custom alignment: LH
116	quality_028	How do you rate the quality of this image?	radio, Required<br>3 Good quality<br>2 Medium quality<br>1 Low quality<br>0 cannot decide<br><br>Custom alignment: LH
117	comment_028	comment to picture 28	text<br>Custom alignment: RH
118	image029	29. Picture	descriptive<br>Field Annotation: Please decide which type you can see in this picture

119	answer029	Which type is picture 29	radio, Required<br>0 no fistula<br>1 bulbar fistula<br>2 prostatic fistula<br>3 bladderneck fistula<br>9 could not decide<br><br>Custom alignment: LH
120	quality_029	How do you rate the quality of this image?	radio, Required<br>3 Good quality<br>2 Medium quality<br>1 Low quality<br>0 cannot decide<br><br>Custom alignment: LH
121	comment_029	comment to picture 29	text<br>Custom alignment: RH
122	image030	30. Picture	descriptive<br>Field Annotation: Please decide which type you can see in this picture
123	answer030	Which type is picture 30	radio, Required<br>0 no fistula<br>1 bulbar fistula<br>2 prostatic fistula<br>3 bladderneck fistula<br>9 could not decide<br><br>Custom alignment: LH
124	quality_030	How do you rate the quality of this image?	radio, Required<br>3 Good quality<br>2 Medium quality<br>1 Low quality<br>0 cannot decide<br><br>Custom alignment: LH
125	comment_030	comment to picture 30	text<br>Custom alignment: RH
126	image031	31. Picture	descriptive<br>Field Annotation: Please decide which type you can see in this picture
127	answer031	Which type is picture 31	radio, Required<br>0 no fistula<br>1 bulbar fistula<br>2 prostatic fistula<br>3 bladderneck fistula<br>9 could not decide<br><br>Custom alignment: LH
128	quality_031	How do you rate the quality of this image?	radio, Required<br>3 Good quality<br>2 Medium quality<br>1 Low quality<br>0 cannot decide<br><br>Custom alignment: LH
129	comment_031	comment to picture 31	text<br>Custom alignment: RH
130	image032	32. Picture	descriptive<br>Field Annotation: Please decide which type you can see in this picture

131	answer032	Which type is picture 32	radio, Required<br>0 no fistula<br>1 bulbar fistula<br>2 prostatic fistula<br>3 bladderneck fistula<br>9 could not decide<br><br>Custom alignment: LH
132	quality_032	How do you rate the quality of this image?	radio, Required<br>3 Good quality<br>2 Medium quality<br>1 Low quality<br>0 cannot decide<br><br>Custom alignment: LH
133	comment_032	comment to picture 32	text<br>Custom alignment: RH
134	image033	33. Picture	descriptive<br>Field Annotation: Please decide which type you can see in this picture
135	answer033	Which type is picture 33	radio, Required<br>0 no fistula<br>1 bulbar fistula<br>2 prostatic fistula<br>3 bladderneck fistula<br>9 could not decide<br><br>Custom alignment: LH
136	quality_033	How do you rate the quality of this image?	radio, Required<br>3 Good quality<br>2 Medium quality<br>1 Low quality<br>0 cannot decide<br><br>Custom alignment: LH
137	comment_033	comment to picture 33	text<br>Custom alignment: RH
138	image034	34. Picture	descriptive<br>Field Annotation: Please decide which type you can see in this picture
139	answer034	Which type is picture 34	radio, Required<br>0 no fistula<br>1 bulbar fistula<br>2 prostatic fistula<br>3 bladderneck fistula<br>9 could not decide<br><br>Custom alignment: LH
140	quality_034	How do you rate the quality of this image?	radio, Required<br>3 Good quality<br>2 Medium quality<br>1 Low quality<br>0 cannot decide<br><br>Custom alignment: LH
141	comment_034	comment to picture 34	text<br>Custom alignment: RH
142	image035	35. Picture	descriptive<br>Field Annotation: Please decide which type you can see in this picture

143	answer035	Which type is picture 35	radio, Required<br>0 no fistula<br>1 bulbar fistula<br>2 prostatic fistula<br>3 bladderneck fistula<br>9 could not decide<br><br>Custom alignment: LH
144	quality_035	How do you rate the quality of this image?	radio, Required<br>3 Good quality<br>2 Medium quality<br>1 Low quality<br>0 cannot decide<br><br>Custom alignment: LH
145	comment_035	comment to picture 35	text<br>Custom alignment: RH
146	image036	36. Picture	descriptive<br>Field Annotation: Please decide which type you can see in this picture
147	answer036	Which type is picture 36	radio, Required<br>0 no fistula<br>1 bulbar fistula<br>2 prostatic fistula<br>3 bladderneck fistula<br>9 could not decide<br><br>Custom alignment: LH
148	quality_036	How do you rate the quality of this image?	radio, Required<br>3 Good quality<br>2 Medium quality<br>1 Low quality<br>0 cannot decide<br><br>Custom alignment: LH
149	comment_036	comment to picture 36	text<br>Custom alignment: RH
150	image037	37. Picture	descriptive<br>Field Annotation: Please decide which type you can see in this picture
151	answer037	Which type is picture 37	radio, Required<br>0 no fistula<br>1 bulbar fistula<br>2 prostatic fistula<br>3 bladderneck fistula<br>9 could not decide<br><br>Custom alignment: LH
152	quality_037	How do you rate the quality of this image?	radio, Required<br>3 Good quality<br>2 Medium quality<br>1 Low quality<br>0 cannot decide<br><br>Custom alignment: LH
153	comment_037	comment to picture 37	text<br>Custom alignment: RH
154	image038	38. Picture	descriptive<br>Field Annotation: Please decide which type you can see in this picture

155	answer038	Which type is picture 38	radio, Required<br>0 no fistula<br>1 bulbar fistula<br>2 prostatic fistula<br>3 bladderneck fistula<br>9 could not decide<br><br>Custom alignment: LH
156	quality_038	How do you rate the quality of this image?	radio, Required<br>3 Good quality<br>2 Medium quality<br>1 Low quality<br>0 cannot decide<br><br>Custom alignment: LH
157	comment_038	comment to picture 38	text<br>Custom alignment: RH
158	image039	39. Picture	descriptive<br>Field Annotation: Please decide which type you can see in this picture
159	answer039	Which type is picture 39	radio, Required<br>0 no fistula<br>1 bulbar fistula<br>2 prostatic fistula<br>3 bladderneck fistula<br>9 could not decide<br><br>Custom alignment: LH
160	quality_039	How do you rate the quality of this image?	radio, Required<br>3 Good quality<br>2 Medium quality<br>1 Low quality<br>0 cannot decide<br><br>Custom alignment: LH
161	comment_039	comment to picture 39	text<br>Custom alignment: RH
162	image040	40. Picture	descriptive<br>Field Annotation: Please decide which type you can see in this picture
163	answer040	Which type is picture 40	radio, Required<br>0 no fistula<br>1 bulbar fistula<br>2 prostatic fistula<br>3 bladderneck fistula<br>9 could not decide<br><br>Custom alignment: LH
164	quality_040	How do you rate the quality of this image?	radio, Required<br>3 Good quality<br>2 Medium quality<br>1 Low quality<br>0 cannot decide<br><br>Custom alignment: LH
165	comment_040	comment to picture 40	text<br>Custom alignment: RH
166	image041	Section Header:<br>41. Picture	descriptive<br>Field Annotation: Please decide which type you can see in this picture

167	answer041	Which type is picture 41	radio, Required<br>0 no fistula<br>1 bulbar fistula<br>2 prostatic fistula<br>3 bladderneck fistula<br>9 could not decide<br><br>Custom alignment: LH
168	quality_041	How do you rate the quality of this image?	radio, Required<br>3 Good quality<br>2 Medium quality<br>1 Low quality<br>0 cannot decide<br><br>Custom alignment: LH
169	comment_041	comment to picture 41	text<br>Custom alignment: RH
170	image042	42. Picture	descriptive<br>Field Annotation: Please decide which type you can see in this picture
171	answer042	Which type is picture 42	radio, Required<br>0 no fistula<br>1 bulbar fistula<br>2 prostatic fistula<br>3 bladderneck fistula<br>9 could not decide<br><br>Custom alignment: LH
172	quality_042	How do you rate the quality of this image?	radio, Required<br>3 Good quality<br>2 Medium quality<br>1 Low quality<br>0 cannot decide<br><br>Custom alignment: LH
173	comment_042	comment to picture 42	text<br>Custom alignment: RH
174	image043	43. Picture	descriptive<br>Field Annotation: Please decide which type you can see in this picture
175	answer043	Which type is picture 43	radio, Required<br>0 no fistula<br>1 bulbar fistula<br>2 prostatic fistula<br>3 bladderneck fistula<br>9 could not decide<br><br>Custom alignment: LH
176	quality_043	How do you rate the quality of this image?	radio, Required<br>3 Good quality<br>2 Medium quality<br>1 Low quality<br>0 cannot decide<br><br>Custom alignment: LH
177	comment_043	comment to picture 43	text<br>Custom alignment: RH
178	image044	44. Picture	descriptive<br>Field Annotation: Please decide which type you can see in this picture

179	answer044	Which type is picture 44	radio, Required<br>0 no fistula<br>1 bulbar fistula<br>2 prostatic fistula<br>3 bladderneck fistula<br>9 could not decide<br><br>Custom alignment: LH
180	quality_044	How do you rate the quality of this image?	radio, Required<br>3 Good quality<br>2 Medium quality<br>1 Low quality<br>0 cannot decide<br><br>Custom alignment: LH
181	comment_044	comment to picture 44	text<br>Custom alignment: RH
182	image045	45. Picture	descriptive<br>Field Annotation: Please decide which type you can see in this picture
183	answer045	Which type is picture 45	radio, Required<br>0 no fistula<br>1 bulbar fistula<br>2 prostatic fistula<br>3 bladderneck fistula<br>9 could not decide<br><br>Custom alignment: LH
184	quality_045	How do you rate the quality of this image?	radio, Required<br>3 Good quality<br>2 Medium quality<br>1 Low quality<br>0 cannot decide<br><br>Custom alignment: LH
185	comment_045	comment to picture 45	text<br>Custom alignment: RH
186	image046	46. Picture	descriptive<br>Field Annotation: Please decide which type you can see in this picture
187	answer046	Which type is picture 46	radio, Required<br>0 no fistula<br>1 bulbar fistula<br>2 prostatic fistula<br>3 bladderneck fistula<br>9 could not decide<br><br>Custom alignment: LH
188	quality_046	How do you rate the quality of this image?	radio, Required<br>3 Good quality<br>2 Medium quality<br>1 Low quality<br>0 cannot decide<br><br>Custom alignment: LH
189	comment_046	comment to picture 46	text<br>Custom alignment: RH
190	image047	47. Picture	descriptive<br>Field Annotation: Please decide which type you can see in this picture

191	answer047	Which type is picture 47	radio, Required<br>0 no fistula<br>1 bulbar fistula<br>2 prostatic fistula<br>3 bladderneck fistula<br>9 could not decide<br><br>Custom alignment: LH
192	quality_047	How do you rate the quality of this image?	radio, Required<br>3 Good quality<br>2 Medium quality<br>1 Low quality<br>0 cannot decide<br><br>Custom alignment: LH
193	comment_047	comment to picture 47	text<br>Custom alignment: RH
194	image048	48. Picture	descriptive<br>Field Annotation: Please decide which type you can see in this picture
195	answer048	Which type is picture 48	radio, Required<br>0 no fistula<br>1 bulbar fistula<br>2 prostatic fistula<br>3 bladderneck fistula<br>9 could not decide<br><br>Custom alignment: LH
196	quality_048	How do you rate the quality of this image?	radio, Required<br>3 Good quality<br>2 Medium quality<br>1 Low quality<br>0 cannot decide<br><br>Custom alignment: LH
197	comment_048	comment to picture 48	text<br>Custom alignment: RH
198	image049	49. Picture	descriptive<br>Field Annotation: Please decide which type you can see in this picture
199	answer049	Which type is picture 49	radio, Required<br>0 no fistula<br>1 bulbar fistula<br>2 prostatic fistula<br>3 bladderneck fistula<br>9 could not decide<br><br>Custom alignment: LH
200	quality_049	How do you rate the quality of this image?	radio, Required<br>3 Good quality<br>2 Medium quality<br>1 Low quality<br>0 cannot decide<br><br>Custom alignment: LH
201	comment_049	comment to picture 49	text<br>Custom alignment: RH
202	image050	50. Picture	descriptive<br>Field Annotation: Please decide which type you can see in this picture

203	answer050	Which type is picture 50	radio, Required<br>0 no fistula<br>1 bulbar fistula<br>2 prostatic fistula<br>3 bladderneck fistula<br>9 could not decide<br><br>Custom alignment: LH
204	quality_050	How do you rate the quality of this image?	radio, Required<br>3 Good quality<br>2 Medium quality<br>1 Low quality<br>0 cannot decide<br><br>Custom alignment: LH
205	comment_050	comment to picture 50	text<br>Custom alignment: RH
206	image051	51. Picture	descriptive<br>Field Annotation: Please decide which type you can see in this picture
207	answer051	Which type is picture 51	radio, Required<br>0 no fistula<br>1 bulbar fistula<br>2 prostatic fistula<br>3 bladderneck fistula<br>9 could not decide<br><br>Custom alignment: LH
208	quality_051	How do you rate the quality of this image?	radio, Required<br>3 Good quality<br>2 Medium quality<br>1 Low quality<br>0 cannot decide<br><br>Custom alignment: LH
209	comment_051	comment to picture 51	text<br>Custom alignment: RH
210	image052	52. Picture	descriptive<br>Field Annotation: Please decide which type you can see in this picture
211	answer052	Which type is picture 52	radio, Required<br>0 no fistula<br>1 bulbar fistula<br>2 prostatic fistula<br>3 bladderneck fistula<br>9 could not decide<br><br>Custom alignment: LH
212	quality_052	How do you rate the quality of this image?	radio, Required<br>3 Good quality<br>2 Medium quality<br>1 Low quality<br>0 cannot decide<br><br>Custom alignment: LH
213	comment_052	comment to picture 52	text<br>Custom alignment: RH
214	image053	53. Picture	descriptive<br>Field Annotation: Please decide which type you can see in this picture

215	answer053	Which type is picture 53	radio, Required<br>0 no fistula<br>1 bulbar fistula<br>2 prostatic fistula<br>3 bladderneck fistula<br>9 could not decide<br><br>Custom alignment: LH
216	quality_053	How do you rate the quality of this image?	radio, Required<br>3 Good quality<br>2 Medium quality<br>1 Low quality<br>0 cannot decide<br><br>Custom alignment: LH
217	comment_053	comment to picture 53	text<br>Custom alignment: RH
218	image054	54. Picture	descriptive<br>Field Annotation: Please decide which type you can see in this picture
219	answer054	Which type is picture 54	radio, Required<br>0 no fistula<br>1 bulbar fistula<br>2 prostatic fistula<br>3 bladderneck fistula<br>9 could not decide<br><br>Custom alignment: LH
220	quality_054	How do you rate the quality of this image?	radio, Required<br>3 Good quality<br>2 Medium quality<br>1 Low quality<br>0 cannot decide<br><br>Custom alignment: LH
221	comment_054	comment to picture 54	text<br>Custom alignment: RH
222	image055	55. Picture	descriptive<br>Field Annotation: Please decide which type you can see in this picture
223	answer055	Which type is picture 55	radio, Required<br>0 no fistula<br>1 bulbar fistula<br>2 prostatic fistula<br>3 bladderneck fistula<br>9 could not decide<br><br>Custom alignment: LH
224	quality_055	How do you rate the quality of this image?	radio, Required<br>3 Good quality<br>2 Medium quality<br>1 Low quality<br>0 cannot decide<br><br>Custom alignment: LH
225	comment_055	comment to picture 55	text<br>Custom alignment: RH
226	image056	56. Picture	descriptive<br>Field Annotation: Please decide which type you can see in this picture

227	answer056	Which type is picture 56	radio, Required<br>0 no fistula<br>1 bulbar fistula<br>2 prostatic fistula<br>3 bladderneck fistula<br>9 could not decide<br><br>Custom alignment: LH
228	quality_056	How do you rate the quality of this image?	radio, Required<br>3 Good quality<br>2 Medium quality<br>1 Low quality<br>0 cannot decide<br><br>Custom alignment: LH
229	comment_056	comment to picture 56	text<br>Custom alignment: RH
230	image057	57. Picture	descriptive<br>Field Annotation: Please decide which type you can see in this picture
231	answer057	Which type is picture 57	radio, Required<br>0 no fistula<br>1 bulbar fistula<br>2 prostatic fistula<br>3 bladderneck fistula<br>9 could not decide<br><br>Custom alignment: LH
232	quality_057	How do you rate the quality of this image?	radio, Required<br>3 Good quality<br>2 Medium quality<br>1 Low quality<br>0 cannot decide<br><br>Custom alignment: LH
233	comment_057	comment to picture 57	text<br>Custom alignment: RH
234	image058	58. Picture	descriptive<br>Field Annotation: Please decide which type you can see in this picture
235	answer058	Which type is picture 58	radio, Required<br>0 no fistula<br>1 bulbar fistula<br>2 prostatic fistula<br>3 bladderneck fistula<br>9 could not decide<br><br>Custom alignment: LH
236	quality_058	How do you rate the quality of this image?	radio, Required<br>3 Good quality<br>2 Medium quality<br>1 Low quality<br>0 cannot decide<br><br>Custom alignment: LH
237	comment_058	comment to picture 58	text<br>Custom alignment: RH
238	image059	59. Picture	descriptive<br>Field Annotation: Please decide which type you can see in this picture

239	answer059	Which type is picture 59	radio, Required<br>0 no fistula<br>1 bulbar fistula<br>2 prostatic fistula<br>3 bladderneck fistula<br>9 could not decide<br><br>Custom alignment: LH
240	quality_059	How do you rate the quality of this image?	radio, Required<br>3 Good quality<br>2 Medium quality<br>1 Low quality<br>0 cannot decide<br><br>Custom alignment: LH
241	comment_059	comment to picture 59	text<br>Custom alignment: RH
242	image060	60. Picture	descriptive<br>Field Annotation: Please decide which type you can see in this picture
243	answer060	Which type is picture 60	radio, Required<br>0 no fistula<br>1 bulbar fistula<br>2 prostatic fistula<br>3 bladderneck fistula<br>9 could not decide<br><br>Custom alignment: LH
244	quality_060	How do you rate the quality of this image?	radio, Required<br>3 Good quality<br>2 Medium quality<br>1 Low quality<br>0 cannot decide<br><br>Custom alignment: LH
245	comment_060	comment to picture 60	text<br>Custom alignment: RH
246	image061	Section Header:<br>61. Picture	descriptive<br>Field Annotation: Please decide which type you can see in this picture
247	answer061	Which type is picture 61	radio, Required<br>0 no fistula<br>1 bulbar fistula<br>2 prostatic fistula<br>3 bladderneck fistula<br>9 could not decide<br><br>Custom alignment: LH
248	quality_061	How do you rate the quality of this image?	radio, Required<br>3 Good quality<br>2 Medium quality<br>1 Low quality<br>0 cannot decide<br><br>Custom alignment: LH
249	comment_061	comment to picture 61	text<br>Custom alignment: RH
250	image062	62. Picture	descriptive<br>Field Annotation: Please decide which type you can see in this picture

251	answer062	Which type is picture 62	radio, Required<br>0 no fistula<br>1 bulbar fistula<br>2 prostatic fistula<br>3 bladderneck fistula<br>9 could not decide<br><br>Custom alignment: LH
252	quality_062	How do you rate the quality of this image?	radio, Required<br>3 Good quality<br>2 Medium quality<br>1 Low quality<br>0 cannot decide<br><br>Custom alignment: LH
253	comment_062	comment to picture 62	text<br>Custom alignment: RH
254	image063	63. Picture	descriptive<br>Field Annotation: Please decide which type you can see in this picture
255	answer063	Which type is picture 63	radio, Required<br>0 no fistula<br>1 bulbar fistula<br>2 prostatic fistula<br>3 bladderneck fistula<br>9 could not decide<br><br>Custom alignment: LH
256	quality_063	How do you rate the quality of this image?	radio, Required<br>3 Good quality<br>2 Medium quality<br>1 Low quality<br>0 cannot decide<br><br>Custom alignment: LH
257	comment_063	comment to picture 63	text<br>Custom alignment: RH
258	image064	64. Picture	descriptive<br>Field Annotation: Please decide which type you can see in this picture
259	answer064	Which type is picture 64	radio, Required<br>0 no fistula<br>1 bulbar fistula<br>2 prostatic fistula<br>3 bladderneck fistula<br>9 could not decide<br><br>Custom alignment: LH
260	quality_064	How do you rate the quality of this image?	radio, Required<br>3 Good quality<br>2 Medium quality<br>1 Low quality<br>0 cannot decide<br><br>Custom alignment: LH
261	comment_064	comment to picture 64	text<br>Custom alignment: RH
262	image065	65. Picture	descriptive<br>Field Annotation: Please decide which type you can see in this picture

263	answer065	Which type is picture 65	radio, Required<br>0 no fistula<br>1 bulbar fistula<br>2 prostatic fistula<br>3 bladderneck fistula<br>9 could not decide<br><br>Custom alignment: LH
264	quality_065	How do you rate the quality of this image?	radio, Required<br>3 Good quality<br>2 Medium quality<br>1 Low quality<br>0 cannot decide<br><br>Custom alignment: LH
265	comment_065	comment to picture 65	text<br>Custom alignment: RH
266	image066	66. Picture	descriptive<br>Field Annotation: Please decide which type you can see in this picture
267	answer066	Which type is picture 66	radio, Required<br>0 no fistula<br>1 bulbar fistula<br>2 prostatic fistula<br>3 bladderneck fistula<br>9 could not decide<br><br>Custom alignment: LH
268	quality_066	How do you rate the quality of this image?	radio, Required<br>3 Good quality<br>2 Medium quality<br>1 Low quality<br>0 cannot decide<br><br>Custom alignment: LH
269	comment_066	comment to picture 66	text<br>Custom alignment: RH
270	image067	67. Picture	descriptive<br>Field Annotation: Please decide which type you can see in this picture
271	answer067	Which type is picture 67	radio, Required<br>0 no fistula<br>1 bulbar fistula<br>2 prostatic fistula<br>3 bladderneck fistula<br>9 could not decide<br><br>Custom alignment: LH
272	quality_067	How do you rate the quality of this image?	radio, Required<br>3 Good quality<br>2 Medium quality<br>1 Low quality<br>0 cannot decide<br><br>Custom alignment: LH
273	comment_067	comment to picture 67	text<br>Custom alignment: RH
274	image068	68. Picture	descriptive<br>Field Annotation: Please decide which type you can see in this picture

275	answer068	Which type is picture 68	radio, Required<br>0 no fistula<br>1 bulbar fistula<br>2 prostatic fistula<br>3 bladderneck fistula<br>9 could not decide<br><br>Custom alignment: LH
276	quality_068	How do you rate the quality of this image?	radio, Required<br>3 Good quality<br>2 Medium quality<br>1 Low quality<br>0 cannot decide<br><br>Custom alignment: LH
277	comment_068	comment to picture 68	text<br>Custom alignment: RH
278	image069	69. Picture	descriptive<br>Field Annotation: Please decide which type you can see in this picture
279	answer069	Which type is picture 69	radio, Required<br>0 no fistula<br>1 bulbar fistula<br>2 prostatic fistula<br>3 bladderneck fistula<br>9 could not decide<br><br>Custom alignment: LH
280	quality_069	How do you rate the quality of this image?	radio, Required<br>3 Good quality<br>2 Medium quality<br>1 Low quality<br>0 cannot decide<br><br>Custom alignment: LH
281	comment_069	comment to picture 69	text<br>Custom alignment: RH
282	image070	70. Picture	descriptive<br>Field Annotation: Please decide which type you can see in this picture
283	answer070	Which type is picture 70	radio, Required<br>0 no fistula<br>1 bulbar fistula<br>2 prostatic fistula<br>3 bladderneck fistula<br>9 could not decide<br><br>Custom alignment: LH
284	quality_070	How do you rate the quality of this image?	radio, Required<br>3 Good quality<br>2 Medium quality<br>1 Low quality<br>0 cannot decide<br><br>Custom alignment: LH
285	comment_070	comment to picture 70	text<br>Custom alignment: RH
286	image071	71. Picture	descriptive<br>Field Annotation: Please decide which type you can see in this picture

287	answer071	Which type is picture 71	radio, Required<br>0 no fistula<br>1 bulbar fistula<br>2 prostatic fistula<br>3 bladderneck fistula<br>9 could not decide<br><br>Custom alignment: LH
288	quality_071	How do you rate the quality of this image?	radio, Required<br>3 Good quality<br>2 Medium quality<br>1 Low quality<br>0 cannot decide<br><br>Custom alignment: LH
289	comment_071	comment to picture 71	text<br>Custom alignment: RH
290	image072	72. Picture	descriptive<br>Field Annotation: Please decide which type you can see in this picture
291	answer072	Which type is picture 72	radio, Required<br>0 no fistula<br>1 bulbar fistula<br>2 prostatic fistula<br>3 bladderneck fistula<br>9 could not decide<br><br>Custom alignment: LH
292	quality_072	How do you rate the quality of this image?	radio, Required<br>3 Good quality<br>2 Medium quality<br>1 Low quality<br>0 cannot decide<br><br>Custom alignment: LH
293	comment_072	comment to picture 72	text<br>Custom alignment: RH
294	image073	73. Picture	descriptive<br>Field Annotation: Please decide which type you can see in this picture
295	answer073	Which type is picture 73	radio, Required<br>0 no fistula<br>1 bulbar fistula<br>2 prostatic fistula<br>3 bladderneck fistula<br>9 could not decide<br><br>Custom alignment: LH
296	quality_073	How do you rate the quality of this image?	radio, Required<br>3 Good quality<br>2 Medium quality<br>1 Low quality<br>0 cannot decide<br><br>Custom alignment: LH
297	comment_073	comment to picture 73	text<br>Custom alignment: RH
298	image074	74. Picture	descriptive<br>Field Annotation: Please decide which type you can see in this picture

299	answer074	Which type is picture 74	radio, Required<br>0 no fistula<br>1 bulbar fistula<br>2 prostatic fistula<br>3 bladderneck fistula<br>9 could not decide<br><br>Custom alignment: LH
300	quality_074	How do you rate the quality of this image?	radio, Required<br>3 Good quality<br>2 Medium quality<br>1 Low quality<br>0 cannot decide<br><br>Custom alignment: LH
301	comment_074	comment to picture 74	text<br>Custom alignment: RH
302	image075	75. Picture	descriptive<br>Field Annotation: Please decide which type you can see in this picture
303	answer075	Which type is picture 75	radio, Required<br>0 no fistula<br>1 bulbar fistula<br>2 prostatic fistula<br>3 bladderneck fistula<br>9 could not decide<br><br>Custom alignment: LH
304	quality_075	How do you rate the quality of this image?	radio, Required<br>3 Good quality<br>2 Medium quality<br>1 Low quality<br>0 cannot decide<br><br>Custom alignment: LH
305	comment_075	comment to picture 75	text<br>Custom alignment: RH
306	image076	76. Picture	descriptive<br>Field Annotation: Please decide which type you can see in this picture
307	answer076	Which type is picture 76	radio, Required<br>0 no fistula<br>1 bulbar fistula<br>2 prostatic fistula<br>3 bladderneck fistula<br>9 could not decide<br><br>Custom alignment: LH
308	quality_076	How do you rate the quality of this image?	radio, Required<br>3 Good quality<br>2 Medium quality<br>1 Low quality<br>0 cannot decide<br><br>Custom alignment: LH
309	comment_076	comment to picture 76	text<br>Custom alignment: RH
310	image077	77. Picture	descriptive<br>Field Annotation: Please decide which type you can see in this picture

311	answer077	Which type is picture 77	radio, Required<br>0 no fistula<br>1 bulbar fistula<br>2 prostatic fistula<br>3 bladderneck fistula<br>9 could not decide<br><br>Custom alignment: LH
312	quality_077	How do you rate the quality of this image?	radio, Required<br>3 Good quality<br>2 Medium quality<br>1 Low quality<br>0 cannot decide<br><br>Custom alignment: LH
313	comment_077	comment to picture 77	text<br>Custom alignment: RH
314	image078	78. Picture	descriptive<br>Field Annotation: Please decide which type you can see in this picture
315	answer078	Which type is picture 78	radio, Required<br>0 no fistula<br>1 bulbar fistula<br>2 prostatic fistula<br>3 bladderneck fistula<br>9 could not decide<br><br>Custom alignment: LH
316	quality_078	How do you rate the quality of this image?	radio, Required<br>3 Good quality<br>2 Medium quality<br>1 Low quality<br>0 cannot decide<br><br>Custom alignment: LH
317	comment_078	comment to picture 78	text<br>Custom alignment: RH
318	image079	79. Picture	descriptive<br>Field Annotation: Please decide which type you can see in this picture
319	answer079	Which type is picture 79	radio, Required<br>0 no fistula<br>1 bulbar fistula<br>2 prostatic fistula<br>3 bladderneck fistula<br>9 could not decide<br><br>Custom alignment: LH
320	quality_079	How do you rate the quality of this image?	radio, Required<br>3 Good quality<br>2 Medium quality<br>1 Low quality<br>0 cannot decide<br><br>Custom alignment: LH
321	comment_079	comment to picture 79	text<br>Custom alignment: RH
322	image080	80. Picture	descriptive<br>Field Annotation: Please decide which type you can see in this picture

323	answer080	Which type is picture 80	radio, Required<br>0 no fistula<br>1 bulbar fistula<br>2 prostatic fistula<br>3 bladderneck fistula<br>9 could not decide<br><br>Custom alignment: LH
324	quality_080	How do you rate the quality of this image?	radio, Required<br>3 Good quality<br>2 Medium quality<br>1 Low quality<br>0 cannot decide<br><br>Custom alignment: LH
325	comment_080	comment to picture 80	text<br>Custom alignment: RH
326	image081	Section Header:<br>81. Picture	descriptive<br>Field Annotation: Please decide which type you can see in this picture
327	answer081	Which type is picture 81	radio, Required<br>0 no fistula<br>1 bulbar fistula<br>2 prostatic fistula<br>3 bladderneck fistula<br>9 could not decide<br><br>Custom alignment: LH
328	quality_081	How do you rate the quality of this image?	radio, Required<br>3 Good quality<br>2 Medium quality<br>1 Low quality<br>0 cannot decide<br><br>Custom alignment: LH
329	comment_081	comment to picture 81	text<br>Custom alignment: RH
330	image082	82. Picture	descriptive<br>Field Annotation: Please decide which type you can see in this picture
331	answer082	Which type is picture 82	radio, Required<br>0 no fistula<br>1 bulbar fistula<br>2 prostatic fistula<br>3 bladderneck fistula<br>9 could not decide<br><br>Custom alignment: LH
332	quality_082	How do you rate the quality of this image?	radio, Required<br>3 Good quality<br>2 Medium quality<br>1 Low quality<br>0 cannot decide<br><br>Custom alignment: LH
333	comment_082	comment to picture 82	text<br>Custom alignment: RH
334	image083	83. Picture	descriptive<br>Field Annotation: Please decide which type you can see in this picture

335	answer083	Which type is picture 83	radio, Required<br>0 no fistula<br>1 bulbar fistula<br>2 prostatic fistula<br>3 bladderneck fistula<br>9 could not decide<br><br>Custom alignment: LH
336	quality_083	How do you rate the quality of this image?	radio, Required<br>3 Good quality<br>2 Medium quality<br>1 Low quality<br>0 cannot decide<br><br>Custom alignment: LH
337	comment_083	comment to picture 83	text<br>Custom alignment: RH
338	image084	84. Picture	descriptive<br>Field Annotation: Please decide which type you can see in this picture
339	answer084	Which type is picture 84	radio, Required<br>0 no fistula<br>1 bulbar fistula<br>2 prostatic fistula<br>3 bladderneck fistula<br>9 could not decide<br><br>Custom alignment: LH
340	quality_084	How do you rate the quality of this image?	radio, Required<br>3 Good quality<br>2 Medium quality<br>1 Low quality<br>0 cannot decide<br><br>Custom alignment: LH
341	comment_084	comment to picture 84	text<br>Custom alignment: RH
342	image085	85. Picture	descriptive<br>Field Annotation: Please decide which type you can see in this picture
343	answer085	Which type is picture 85	radio, Required<br>0 no fistula<br>1 bulbar fistula<br>2 prostatic fistula<br>3 bladderneck fistula<br>9 could not decide<br><br>Custom alignment: LH
344	quality_085	How do you rate the quality of this image?	radio, Required<br>3 Good quality<br>2 Medium quality<br>1 Low quality<br>0 cannot decide<br><br>Custom alignment: LH
345	comment_085	comment to picture 85	text<br>Custom alignment: RH
346	image086	86. Picture	descriptive<br>Field Annotation: Please decide which type you can see in this picture

347	answer086	Which type is picture 86	radio, Required<br>0 no fistula<br>1 bulbar fistula<br>2 prostatic fistula<br>3 bladderneck fistula<br>9 could not decide<br><br>Custom alignment: LH
348	quality_086	How do you rate the quality of this image?	radio, Required<br>3 Good quality<br>2 Medium quality<br>1 Low quality<br>0 cannot decide<br><br>Custom alignment: LH
349	comment_086	comment to picture 86	text<br>Custom alignment: RH
350	image087	87. Picture	descriptive<br>Field Annotation: Please decide which type you can see in this picture
351	answer087	Which type is picture 87	radio, Required<br>0 no fistula<br>1 bulbar fistula<br>2 prostatic fistula<br>3 bladderneck fistula<br>9 could not decide<br><br>Custom alignment: LH
352	quality_087	How do you rate the quality of this image?	radio, Required<br>3 Good quality<br>2 Medium quality<br>1 Low quality<br>0 cannot decide<br><br>Custom alignment: LH
353	comment_087	comment to picture 87	text<br>Custom alignment: RH
354	image088	88. Picture	descriptive<br>Field Annotation: Please decide which type you can see in this picture
355	answer088	Which type is picture 88	radio, Required<br>0 no fistula<br>1 bulbar fistula<br>2 prostatic fistula<br>3 bladderneck fistula<br>9 could not decide<br><br>Custom alignment: LH
356	quality_088	How do you rate the quality of this image?	radio, Required<br>3 Good quality<br>2 Medium quality<br>1 Low quality<br>0 cannot decide<br><br>Custom alignment: LH
357	comment_088	comment to picture 88	text<br>Custom alignment: RH
358	image089	89. Picture	descriptive<br>Field Annotation: Please decide which type you can see in this picture

359	answer089	Which type is picture 89	radio, Required<br>0 no fistula<br>1 bulbar fistula<br>2 prostatic fistula<br>3 bladderneck fistula<br>9 could not decide<br><br>Custom alignment: LH
360	quality_089	How do you rate the quality of this image?	radio, Required<br>3 Good quality<br>2 Medium quality<br>1 Low quality<br>0 cannot decide<br><br>Custom alignment: LH
361	comment_089	comment to picture 89	text<br>Custom alignment: RH
362	image090	90. Picture	descriptive<br>Field Annotation: Please decide which type you can see in this picture
363	answer090	Which type is picture 90	radio, Required<br>0 no fistula<br>1 bulbar fistula<br>2 prostatic fistula<br>3 bladderneck fistula<br>9 could not decide<br><br>Custom alignment: LH
364	quality_090	How do you rate the quality of this image?	radio, Required<br>3 Good quality<br>2 Medium quality<br>1 Low quality<br>0 cannot decide<br><br>Custom alignment: LH
365	comment_090	comment to picture 90	text<br>Custom alignment: RH
366	image091	91. Picture	descriptive<br>Field Annotation: Please decide which type you can see in this picture
367	answer091	Which type is picture 91	radio, Required<br>0 no fistula<br>1 bulbar fistula<br>2 prostatic fistula<br>3 bladderneck fistula<br>9 could not decide<br><br>Custom alignment: LH
368	quality_091	How do you rate the quality of this image?	radio, Required<br>3 Good quality<br>2 Medium quality<br>1 Low quality<br>0 cannot decide<br><br>Custom alignment: LH
369	comment_091	comment to picture 91	text<br>Custom alignment: RH
370	image092	92. Picture	descriptive<br>Field Annotation: Please decide which type you can see in this picture

371	answer092	Which type is picture 92	radio, Required<br>0 no fistula<br>1 bulbar fistula<br>2 prostatic fistula<br>3 bladderneck fistula<br>9 could not decide<br><br>Custom alignment: LH
372	quality_092	How do you rate the quality of this image?	radio, Required<br>3 Good quality<br>2 Medium quality<br>1 Low quality<br>0 cannot decide<br><br>Custom alignment: LH
373	comment_092	comment to picture 92	text<br>Custom alignment: RH
374	image093	93. Picture	descriptive<br>Field Annotation: Please decide which type you can see in this picture
375	answer093	Which type is picture 93	radio, Required<br>0 no fistula<br>1 bulbar fistula<br>2 prostatic fistula<br>3 bladderneck fistula<br>9 could not decide<br><br>Custom alignment: LH
376	quality_093	How do you rate the quality of this image?	radio, Required<br>3 Good quality<br>2 Medium quality<br>1 Low quality<br>0 cannot decide<br><br>Custom alignment: LH
377	comment_093	comment to picture 93	text<br>Custom alignment: RH
378	image094	94. Picture	descriptive<br>Field Annotation: Please decide which type you can see in this picture
379	answer094	Which type is picture 94	radio, Required<br>0 no fistula<br>1 bulbar fistula<br>2 prostatic fistula<br>3 bladderneck fistula<br>9 could not decide<br><br>Custom alignment: LH
380	quality_094	How do you rate the quality of this image?	radio, Required<br>3 Good quality<br>2 Medium quality<br>1 Low quality<br>0 cannot decide<br><br>Custom alignment: LH
381	comment_094	comment to picture 94	text<br>Custom alignment: RH
382	image095	95. Picture	descriptive<br>Field Annotation: Please decide which type you can see in this picture

383	answer095	Which type is picture 95	radio, Required<br>0 no fistula<br>1 bulbar fistula<br>2 prostatic fistula<br>3 bladderneck fistula<br>9 could not decide<br><br>Custom alignment: LH
384	quality_095	How do you rate the quality of this image?	radio, Required<br>3 Good quality<br>2 Medium quality<br>1 Low quality<br>0 cannot decide<br><br>Custom alignment: LH
385	comment_095	comment to picture 95	text<br>Custom alignment: RH
386	image096	96. Picture	descriptive<br>Field Annotation: Please decide which type you can see in this picture
387	answer096	Which type is picture 96	radio, Required<br>0 no fistula<br>1 bulbar fistula<br>2 prostatic fistula<br>3 bladderneck fistula<br>9 could not decide<br><br>Custom alignment: LH
388	quality_096	How do you rate the quality of this image?	radio, Required<br>3 Good quality<br>2 Medium quality<br>1 Low quality<br>0 cannot decide<br><br>Custom alignment: LH
389	comment_096	comment to picture 96	text<br>Custom alignment: RH
390	image097	97. Picture	descriptive<br>Field Annotation: Please decide which type you can see in this picture
391	answer097	Which type is picture 97	radio, Required<br>0 no fistula<br>1 bulbar fistula<br>2 prostatic fistula<br>3 bladderneck fistula<br>9 could not decide<br><br>Custom alignment: LH
392	quality_097	How do you rate the quality of this image?	radio, Required<br>3 Good quality<br>2 Medium quality<br>1 Low quality<br>0 cannot decide<br><br>Custom alignment: LH
393	comment_097	comment to picture 97	text<br>Custom alignment: RH
394	image098	98. Picture	descriptive<br>Field Annotation: Please decide which type you can see in this picture

395	answer098	Which type is picture 98	radio, Required<br>0 no fistula<br>1 bulbar fistula<br>2 prostatic fistula<br>3 bladderneck fistula<br>9 could not decide<br><br>Custom alignment: LH
396	quality_098	How do you rate the quality of this image?	radio, Required<br>3 Good quality<br>2 Medium quality<br>1 Low quality<br>0 cannot decide<br><br>Custom alignment: LH
397	comment_098	comment to picture 98	text<br>Custom alignment: RH
398	image099	99. Picture	descriptive<br>Field Annotation: Please decide which type you can see in this picture
399	answer099	Which type is picture 99	radio, Required<br>0 no fistula<br>1 bulbar fistula<br>2 prostatic fistula<br>3 bladderneck fistula<br>9 could not decide<br><br>Custom alignment: LH
400	quality_099	How do you rate the quality of this image?	radio, Required<br>3 Good quality<br>2 Medium quality<br>1 Low quality<br>0 cannot decide<br><br>Custom alignment: LH
401	comment_099	comment to picture 99	text<br>Custom alignment: RH
402	image100	100. Picture	descriptive<br>Field Annotation: Please decide which type you can see in this picture
403	answer100	Which type is picture 100	radio, Required<br>0 no fistula<br>1 bulbar fistula<br>2 prostatic fistula<br>3 bladderneck fistula<br>9 could not decide<br><br>Custom alignment: LH
404	quality_100	How do you rate the quality of this image?	radio, Required<br>3 Good quality<br>2 Medium quality<br>1 Low quality<br>0 cannot decide<br><br>Custom alignment: LH
405	comment_100	comment to picture 100	text<br>Custom alignment: RH
406	image101	Section Header:<br>101. Picture	descriptive<br>Field Annotation: Please decide which type you can see in this picture

407	answer101	Which type is picture 101	radio, Required<br>0 no fistula<br>1 bulbar fistula<br>2 prostatic fistula<br>3 bladderneck fistula<br>9 could not decide<br><br>Custom alignment: LH
408	quality_101	How do you rate the quality of this image?	radio, Required<br>3 Good quality<br>2 Medium quality<br>1 Low quality<br>0 cannot decide<br><br>Custom alignment: LH
409	comment_101	comment to picture 101	text<br>Custom alignment: RH
410	image102	102. Picture	descriptive<br>Field Annotation: Please decide which type you can see in this picture
411	answer102	Which type is picture 102	radio, Required<br>0 no fistula<br>1 bulbar fistula<br>2 prostatic fistula<br>3 bladderneck fistula<br>9 could not decide<br><br>Custom alignment: LH
412	quality_102	How do you rate the quality of this image?	radio, Required<br>3 Good quality<br>2 Medium quality<br>1 Low quality<br>0 cannot decide<br><br>Custom alignment: LH
413	comment_102	comment to picture 102	text<br>Custom alignment: RH
414	image103	103. Picture	descriptive<br>Field Annotation: Please decide which type you can see in this picture
415	answer103	Which type is picture 103	radio, Required<br>0 no fistula<br>1 bulbar fistula<br>2 prostatic fistula<br>3 bladderneck fistula<br>9 could not decide<br><br>Custom alignment: LH
416	quality_103	How do you rate the quality of this image?	radio, Required<br>3 Good quality<br>2 Medium quality<br>1 Low quality<br>0 cannot decide<br><br>Custom alignment: LH
417	comment_103	comment to picture 103	text<br>Custom alignment: RH
418	image104	104. Picture	descriptive<br>Field Annotation: Please decide which type you can see in this picture

419	answer104	Which type is picture 104	radio, Required<br>0 no fistula<br>1 bulbar fistula<br>2 prostatic fistula<br>3 bladderneck fistula<br>9 could not decide<br><br>Custom alignment: LH
420	quality_104	How do you rate the quality of this image?	radio, Required<br>3 Good quality<br>2 Medium quality<br>1 Low quality<br>0 cannot decide<br><br>Custom alignment: LH
421	comment_104	comment to picture 104	text<br>Custom alignment: RH
422	image105	105. Picture	descriptive<br>Field Annotation: Please decide which type you can see in this picture
423	answer105	Which type is picture 105	radio, Required<br>0 no fistula<br>1 bulbar fistula<br>2 prostatic fistula<br>3 bladderneck fistula<br>9 could not decide<br><br>Custom alignment: LH
424	quality_105	How do you rate the quality of this image?	radio, Required<br>3 Good quality<br>2 Medium quality<br>1 Low quality<br>0 cannot decide<br><br>Custom alignment: LH
425	comment_105	comment to picture 105	text<br>Custom alignment: RH
426	image106	106. Picture	descriptive<br>Field Annotation: Please decide which type you can see in this picture
427	answer106	Which type is picture 106	radio, Required<br>0 no fistula<br>1 bulbar fistula<br>2 prostatic fistula<br>3 bladderneck fistula<br>9 could not decide<br><br>Custom alignment: LH
428	quality_106	How do you rate the quality of this image?	radio, Required<br>3 Good quality<br>2 Medium quality<br>1 Low quality<br>0 cannot decide<br><br>Custom alignment: LH
429	comment_106	comment to picture 106	text<br>Custom alignment: RH
430	image107	107. Picture	descriptive<br>Field Annotation: Please decide which type you can see in this picture

431	answer107	Which type is picture 107	radio, Required<br>0 no fistula<br>1 bulbar fistula<br>2 prostatic fistula<br>3 bladderneck fistula<br>9 could not decide<br><br>Custom alignment: LH
432	quality_107	How do you rate the quality of this image?	radio, Required<br>3 Good quality<br>2 Medium quality<br>1 Low quality<br>0 cannot decide<br><br>Custom alignment: LH
433	comment_107	comment to picture 107	text<br>Custom alignment: RH
434	image108	108. Picture	descriptive<br>Field Annotation: Please decide which type you can see in this picture
435	answer108	Which type is picture 108	radio, Required<br>0 no fistula<br>1 bulbar fistula<br>2 prostatic fistula<br>3 bladderneck fistula<br>9 could not decide<br><br>Custom alignment: LH
436	quality_108	How do you rate the quality of this image?	radio, Required<br>3 Good quality<br>2 Medium quality<br>1 Low quality<br>0 cannot decide<br><br>Custom alignment: LH
437	comment_108	comment to picture 108	text<br>Custom alignment: RH
438	image109	109. Picture	descriptive<br>Field Annotation: Please decide which type you can see in this picture
439	answer109	Which type is picture 109	radio, Required<br>0 no fistula<br>1 bulbar fistula<br>2 prostatic fistula<br>3 bladderneck fistula<br>9 could not decide<br><br>Custom alignment: LH
440	quality_109	How do you rate the quality of this image?	radio, Required<br>3 Good quality<br>2 Medium quality<br>1 Low quality<br>0 cannot decide<br><br>Custom alignment: LH
441	comment_109	comment to picture 109	text<br>Custom alignment: RH
442	image110	110. Picture	descriptive<br>Field Annotation: Please decide which type you can see in this picture

443	answer110	Which type is picture 110	radio, Required<br>0 no fistula<br>1 bulbar fistula<br>2 prostatic fistula<br>3 bladderneck fistula<br>9 could not decide<br><br>Custom alignment: LH
444	quality_110	How do you rate the quality of this image?	radio, Required<br>3 Good quality<br>2 Medium quality<br>1 Low quality<br>0 cannot decide<br><br>Custom alignment: LH
445	comment_110	comment to picture 110	text<br>Custom alignment: RH
446	image111	111. Picture	descriptive<br>Field Annotation: Please decide which type you can see in this picture
447	answer111	Which type is picture 111	radio, Required<br>0 no fistula<br>1 bulbar fistula<br>2 prostatic fistula<br>3 bladderneck fistula<br>9 could not decide<br><br>Custom alignment: LH
448	quality_111	How do you rate the quality of this image?	radio, Required<br>3 Good quality<br>2 Medium quality<br>1 Low quality<br>0 cannot decide<br><br>Custom alignment: LH
449	comment_111	comment to picture 111	text<br>Custom alignment: RH
450	image112	112. Picture	descriptive<br>Field Annotation: Please decide which type you can see in this picture
451	answer112	Which type is picture 112	radio, Required<br>0 no fistula<br>1 bulbar fistula<br>2 prostatic fistula<br>3 bladderneck fistula<br>9 could not decide<br><br>Custom alignment: LH
452	quality_112	How do you rate the quality of this image?	radio, Required<br>3 Good quality<br>2 Medium quality<br>1 Low quality<br>0 cannot decide<br><br>Custom alignment: LH
453	comment_112	comment to picture 112	text<br>Custom alignment: RH
454	image113	113. Picture	descriptive<br>Field Annotation: Please decide which type you can see in this picture

455	answer113	Which type is picture 113	radio, Required<br>0 no fistula<br>1 bulbar fistula<br>2 prostatic fistula<br>3 bladderneck fistula<br>9 could not decide<br><br>Custom alignment: LH
456	quality_113	How do you rate the quality of this image?	radio, Required<br>3 Good quality<br>2 Medium quality<br>1 Low quality<br>0 cannot decide<br><br>Custom alignment: LH
457	comment_113	comment to picture 113	text<br>Custom alignment: RH
458	image114	114. Picture	descriptive<br>Field Annotation: Please decide which type you can see in this picture
459	answer114	Which type is picture 114	radio, Required<br>0 no fistula<br>1 bulbar fistula<br>2 prostatic fistula<br>3 bladderneck fistula<br>9 could not decide<br><br>Custom alignment: LH
460	quality_114	How do you rate the quality of this image?	radio, Required<br>3 Good quality<br>2 Medium quality<br>1 Low quality<br>0 cannot decide<br><br>Custom alignment: LH
461	comment_114	comment to picture 114	text<br>Custom alignment: RH
462	image115	115. Picture	descriptive<br>Field Annotation: Please decide which type you can see in this picture
463	answer115	Which type is picture 115	radio, Required<br>0 no fistula<br>1 bulbar fistula<br>2 prostatic fistula<br>3 bladderneck fistula<br>9 could not decide<br><br>Custom alignment: LH
464	quality_115	How do you rate the quality of this image?	radio, Required<br>3 Good quality<br>2 Medium quality<br>1 Low quality<br>0 cannot decide<br><br>Custom alignment: LH
465	comment_115	comment to picture 115	text<br>Custom alignment: RH
466	image116	116. Picture	descriptive<br>Field Annotation: Please decide which type you can see in this picture

467	answer116	Which type is picture 116	radio, Required<br>0 no fistula<br>1 bulbar fistula<br>2 prostatic fistula<br>3 bladderneck fistula<br>9 could not decide<br><br>Custom alignment: LH
468	quality_116	How do you rate the quality of this image?	radio, Required<br>3 Good quality<br>2 Medium quality<br>1 Low quality<br>0 cannot decide<br><br>Custom alignment: LH
469	comment_116	comment to picture 116	text<br>Custom alignment: RH
470	image117	117. Picture	descriptive<br>Field Annotation: Please decide which type you can see in this picture
471	answer117	Which type is picture 117	radio, Required<br>0 no fistula<br>1 bulbar fistula<br>2 prostatic fistula<br>3 bladderneck fistula<br>9 could not decide<br><br>Custom alignment: LH
472	quality_117	How do you rate the quality of this image?	radio, Required<br>3 Good quality<br>2 Medium quality<br>1 Low quality<br>0 cannot decide<br><br>Custom alignment: LH
473	comment_117	comment to picture 117	text<br>Custom alignment: RH
474	image118	118. Picture	descriptive<br>Field Annotation: Please decide which type you can see in this picture
475	answer118	Which type is picture 118	radio, Required<br>0 no fistula<br>1 bulbar fistula<br>2 prostatic fistula<br>3 bladderneck fistula<br>9 could not decide<br><br>Custom alignment: LH
476	quality_118	How do you rate the quality of this image?	radio, Required<br>3 Good quality<br>2 Medium quality<br>1 Low quality<br>0 cannot decide<br><br>Custom alignment: LH
477	comment_118	comment to picture 118	text<br>Custom alignment: RH
478	image119	119. Picture	descriptive<br>Field Annotation: Please decide which type you can see in this picture

479	answer119	Which type is picture 119	radio, Required<br>0 no fistula<br>1 bulbar fistula<br>2 prostatic fistula<br>3 bladderneck fistula<br>9 could not decide<br><br>Custom alignment: LH
480	quality_119	How do you rate the quality of this image?	radio, Required<br>3 Good quality<br>2 Medium quality<br>1 Low quality<br>0 cannot decide<br><br>Custom alignment: LH
481	comment_119	comment to picture 119	text<br>Custom alignment: RH
482	image120	120. Picture	descriptive<br>Field Annotation: Please decide which type you can see in this picture
483	answer120	Which type is picture 120	radio, Required<br>0 no fistula<br>1 bulbar fistula<br>2 prostatic fistula<br>3 bladderneck fistula<br>9 could not decide<br><br>Custom alignment: LH
484	quality_120	How do you rate the quality of this image?	radio, Required<br>3 Good quality<br>2 Medium quality<br>1 Low quality<br>0 cannot decide<br><br>Custom alignment: LH
485	comment_120	comment to picture 120	text<br>Custom alignment: RH
486	image121	121. Picture	descriptive<br>Field Annotation: Please decide which type you can see in this picture
487	answer121	Which type is picture 121	radio, Required<br>0 no fistula<br>1 bulbar fistula<br>2 prostatic fistula<br>3 bladderneck fistula<br>9 could not decide<br><br>Custom alignment: LH
488	quality_121	How do you rate the quality of this image?	radio, Required<br>3 Good quality<br>2 Medium quality<br>1 Low quality<br>0 cannot decide<br><br>Custom alignment: LH
489	comment_121	comment to picture 121	text<br>Custom alignment: RH
490	image122	122. Picture	descriptive<br>Field Annotation: Please decide which type you can see in this picture

491	answer122	Which type is picture 122	radio, Required<br>0 no fistula<br>1 bulbar fistula<br>2 prostatic fistula<br>3 bladderneck fistula<br>9 could not decide<br><br>Custom alignment: LH
492	quality_122	How do you rate the quality of this image?	radio, Required<br>3 Good quality<br>2 Medium quality<br>1 Low quality<br>0 cannot decide<br><br>Custom alignment: LH
493	comment_122	comment to picture 122	text<br>Custom alignment: RH
494	image123	123. Picture	descriptive<br>Field Annotation: Please decide which type you can see in this picture
495	answer123	Which type is picture 123	radio, Required<br>0 no fistula<br>1 bulbar fistula<br>2 prostatic fistula<br>3 bladderneck fistula<br>9 could not decide<br><br>Custom alignment: LH
496	quality_123	How do you rate the quality of this image?	radio, Required<br>3 Good quality<br>2 Medium quality<br>1 Low quality<br>0 cannot decide<br><br>Custom alignment: LH
497	comment_123	comment to picture 123	text<br>Custom alignment: RH
498	image124	124. Picture	descriptive<br>Field Annotation: Please decide which type you can see in this picture
499	answer124	Which type is picture 124	radio, Required<br>0 no fistula<br>1 bulbar fistula<br>2 prostatic fistula<br>3 bladderneck fistula<br>9 could not decide<br><br>Custom alignment: LH
500	quality_124	How do you rate the quality of this image?	radio, Required<br>3 Good quality<br>2 Medium quality<br>1 Low quality<br>0 cannot decide<br><br>Custom alignment: LH
501	comment_124	comment to picture 124	text<br>Custom alignment: RH
502	image125	125. Picture	descriptive<br>Field Annotation: Please decide which type you can see in this picture

503	answer125	Which type is picture 125	radio, Required<br>0 no fistula<br>1 bulbar fistula<br>2 prostatic fistula<br>3 bladderneck fistula<br>9 could not decide<br><br>Custom alignment: LH
504	quality_125	How do you rate the quality of this image?	radio, Required<br>3 Good quality<br>2 Medium quality<br>1 Low quality<br>0 cannot decide<br><br>Custom alignment: LH
505	comment_125	comment to picture 125	text<br>Custom alignment: RH
506	image126	126. Picture	descriptive<br>Field Annotation: Please decide which type you can see in this picture
507	answer126	Which type is picture 126	radio, Required<br>0 no fistula<br>1 bulbar fistula<br>2 prostatic fistula<br>3 bladderneck fistula<br>9 could not decide<br><br>Custom alignment: LH
508	quality_126	How do you rate the quality of this image?	radio, Required<br>3 Good quality<br>2 Medium quality<br>1 Low quality<br>0 cannot decide<br><br>Custom alignment: LH
509	comment_126	comment to picture 126	text<br>Custom alignment: RH
510	image127	127. Picture	descriptive<br>Field Annotation: Please decide which type you can see in this picture
511	answer127	Which type is picture 127	radio, Required<br>0 no fistula<br>1 bulbar fistula<br>2 prostatic fistula<br>3 bladderneck fistula<br>9 could not decide<br><br>Custom alignment: LH
512	quality_127	How do you rate the quality of this image?	radio, Required<br>3 Good quality<br>2 Medium quality<br>1 Low quality<br>0 cannot decide<br><br>Custom alignment: LH
513	comment_127	comment to picture 127	text<br>Custom alignment: RH
514	image128	128. Picture	descriptive<br>Field Annotation: Please decide which type you can see in this picture

515	answer128	Which type is picture 128	radio, Required<br>0 no fistula<br>1 bulbar fistula<br>2 prostatic fistula<br>3 bladderneck fistula<br>9 could not decide<br><br>Custom alignment: LH
516	quality_128	How do you rate the quality of this image?	radio, Required<br>3 Good quality<br>2 Medium quality<br>1 Low quality<br>0 cannot decide<br><br>Custom alignment: LH
517	comment_128	comment to picture 128	text<br>Custom alignment: RH
518	image129	129. Picture	descriptive<br>Field Annotation: Please decide which type you can see in this picture
519	answer129	Which type is picture 129	radio, Required<br>0 no fistula<br>1 bulbar fistula<br>2 prostatic fistula<br>3 bladderneck fistula<br>9 could not decide<br><br>Custom alignment: LH
520	quality_129	How do you rate the quality of this image?	radio, Required<br>3 Good quality<br>2 Medium quality<br>1 Low quality<br>0 cannot decide<br><br>Custom alignment: LH
521	comment_129	comment to picture 129	text<br>Custom alignment: RH
522	image130	130. Picture	descriptive<br>Field Annotation: Please decide which type you can see in this picture
523	answer130	Which type is picture 130	radio, Required<br>0 no fistula<br>1 bulbar fistula<br>2 prostatic fistula<br>3 bladderneck fistula<br>9 could not decide<br><br>Custom alignment: LH
524	quality_130	How do you rate the quality of this image?	radio, Required<br>3 Good quality<br>2 Medium quality<br>1 Low quality<br>0 cannot decide<br><br>Custom alignment: LH
525	comment_130	comment to picture 130	text<br>Custom alignment: RH
526	comment_final	Do you want to leave any final comment?	notes<br>Custom alignment: RH
527	duration	How many hours and minutes took the rating?	text (time, Min: 00:15, Max: 20:00)
528	email	Please enter the email of you radiologist, which want to be invited for the radiological part of this study to score these images. If there is no radiologist, just write "no radiologist".	text

529	urethral_fistula_images_with_quality_complete	Section Header: *Form Status*<br>Complete?	dropdown<br>0 Incomplete<br>1 Unverified<br>2 Complete
